# Inflammatory conversion of quiescent osteoblasts by metastatic breast cancer cells through pERK1/2 aggravates cancer-induced bone destruction

**DOI:** 10.1038/s41413-021-00158-w

**Published:** 2021-09-29

**Authors:** Jungho Back, Minh Nam Nguyen, Lu Li, Saelim Lee, Inkyu Lee, Fancheng Chen, Lauren Gillinov, Yeon-Ho Chung, Kareme D. Alder, Hyuk-Kwon Kwon, Kristin E. Yu, Christopher M. Dussik, Zichen Hao, Michael J. Flores, Yoseph Kim, Izuchukwu K. Ibe, Alana M. Munger, Sung Wook Seo, Francis Y. Lee

**Affiliations:** 1 Department of Orthopædics & Rehabilitation, Yale University, School of Medicine, New Haven, CT USA; 2 Research Center for Genetics and Reproductive Health, School of Medicine, Vietnam National University Ho Chi Minh City, Linh Trung Ward, Thu Duc District, Ho Chi Minh City, Vietnam; 3 Department of Rehabilitation Medicine, Renji Hospital, School of Medicine, Shanghai JiaoTong University, Shanghai, China; 4 College of Medicine, Dankook University, Cheonan, Republic of Korea; 5 Department of Life Science, Chung-Ang University, Seoul, Republic of Korea; 6 Shanghai Medical College, Fudan University, Shanghai City, China; 7 Department of Emergency & Trauma, Changhai Hospital, Navy Medical University, Shanghai, China; 8 Biomedical Engineering, Johns Hopkins University, Baltimore, MD USA; 9 Department of Orthopedic Surgery, Samsung Medical Center, Sungkyunkwan University School of Medicine, Seoul, Gangnam-gu Republic of Korea

**Keywords:** Bone, Bone cancer

## Abstract

Disruption of bone homeostasis caused by metastatic osteolytic breast cancer cells increases inflammatory osteolysis and decreases bone formation, thereby predisposing patients to pathological fracture and cancer growth. Alteration of osteoblast function induces skeletal diseases due to the disruption of bone homeostasis. We observed increased activation of pERK1/2 in osteolytic breast cancer cells and osteoblasts in human pathological specimens with aggressive osteolytic breast cancer metastases. We confirmed that osteolytic breast cancers with high expression of pERK1/2 disrupt bone homeostasis via osteoblastic ERK1/2 activation at the bone-breast cancer interface. The process of inflammatory osteolysis modulates ERK1/2 activation in osteoblasts and breast cancer cells through dominant-negative MEK1 expression and constitutively active MEK1 expression to promote cancer growth within bone. Trametinib, an FDA-approved MEK inhibitor, not only reduced breast cancer-induced bone destruction but also dramatically reduced cancer growth in bone by inhibiting the inflammatory skeletal microenvironment. Taken together, these findings suggest that ERK1/2 activation in both breast cancer cells and osteoblasts is required for osteolytic breast cancer-induced inflammatory osteolysis and that ERK1/2 pathway inhibitors may represent a promising adjuvant therapy for patients with aggressive osteolytic breast cancers by altering the shared cancer and bone microenvironment.

## Introduction

Breast cancer is the most diagnosed cancer in women. More than 271 270 new cases were diagnosed in the United States in 2019, resulting in more than 42 260 deaths.^1^ The development of breast cancer is further complicated by the disease’s high metastatic potential. Up to 70% of women diagnosed with breast cancer will develop metastatic bone disease.^2^ The skeleton is the largest tumor reservoir in the body, allowing both tumor recurrence and the development of tumor-related skeletal events such as local bone destruction, debilitating bone pain, pathologic fractures, compression of neurovascular bundles, and paralysis. These sequelae increase disease-related morbidity and mortality.^3–7^

Intriguingly, some breast cancer cells (BCCs) display excellent osteotropism and grow well in the skeletal compartment. It has yet to be ascertained how select BCCs adapt to the skeletal environment and expand in the physically rigid structure of bone. Although BCCs cannot directly resorb bone, bone metastases in the setting of breast cancer are frequently osteolytic.^8,9^ Osteoclastogenesis is paradoxically facilitated by osteoblasts through the production of receptor activator of NF-κB ligand (RANKL) and osteoclastogenic cytokines.^10^ Skeletal remodeling is tightly regulated through the actions of osteoclasts and osteoblasts. The pathogenesis of malignancy and its sequelae are caused by imbalances in normal homeostatic mechanisms. By disrupting the homeostasis between these cells, BCCs promote osteolysis while simultaneously inhibiting bone formation. The mechanism by which BCCs disrupt this tightly regulated process is through the secretion of factors such as parathyroid hormone-related protein (PTHrP), interleukin 6 (IL-6), JAGGED1 (JAG1), and vascular endothelial growth factor (VEGF), which alter the bone microenvironment and induce osteoclastogenesis at the expense of osteoblastic activity.^11–13^ However, the manner in which these factors interact with osteoblasts to impair anabolism and invoke their osteoclastogenic potential is poorly understood. An improved understanding of this process is essential for the development of novel treatment modalities to manage skeletal pathologies in metastatic breast cancer.

Our previous research showed that ERK1/2 activation in cancer cells promotes inflammatory osteolysis in bone.^14,15^ In the present study, we found that ERK1/2 is highly activated in human osteolytic cancer cell lines, but ERK1/2 is less activated in human osteoblastic cancer lines. Furthermore, interactions between BCCs and osteoblasts via ERK1/2 activation mediate the phenotypic switching of osteoblasts from an anabolic to catabolic state, which is required for BCC proliferation and destruction of bone. Signaling through mitogen-activated protein kinase (MAPK or pERK1/2) is crucial to cancer cell proliferation and differentiation^16^ and inflammatory cytokine production via the RAS-RAF-MEK-ERK1/2 cascade.^17^ With respect to the downstream effects of MAPK signaling on bone remodeling, previous studies have shown that [1] proinflammatory states are osteoclastogenic,^18^ [2] MEK inhibitors enhance osteoblastic differentiation,^19^ although constitutive activation of MEK-1 hinders osteoblastic differentiation,^20^ and [3] ERK1/2 inactivation significantly reduces osteoblast RANKL expression.^21^

Therapies targeting RAF/MEK/ERK expression and/or signaling have been approved for the treatment of unresectable or metastatic melanoma associated with BRAF V600E or V600K mutations. Since MEK mutations are rare in human cancer and MEK1 inhibitors show normal tissue toxicity in cancer therapy,^16^ MEK inhibitors are commonly used clinically in combination with BRAF inhibitors. Despite significant therapeutic effects, MAPK reactivation by acquired drug resistance-related mutations was observed in BRAF^V600^-mutant melanoma patients treated with dabrafenib plus trametinib.^22^ Vascular endothelial growth factor (VEGF) inhibitors do not directly induce death in cancer cells but are FDA approved for cancer treatment due to their role in blocking angiogenesis, thereby inhibiting cell growth.^23^ The anti-osteoclastogenic drugs bisphosphonate and denosumab have already been approved as adjuvant therapies for metastatic breast cancers to bone even though these drugs did not show cytotoxic effects on cancer cells.^24^ Even if BCCs acquire drug resistance to MEK1 inhibitors, these drugs may exert therapeutic effects through bone cells. Given this knowledge, the RAS-RAF-MEK-ERK1/2 pathway was investigated as a therapeutic target for bone loss secondary to metastatic breast cancer.

We posit that the metastasis of BCCs to bone alters the bone microenvironment and that suppression of these changes represents a viable therapeutic strategy. An improved understanding of the microenvironmental changes caused by cancer cells is pivotal to the identification and development of new, effective treatments for skeletal metastases in advanced breast cancer.

## Results

### Aggressive osteolytic BCCs highly express pERK1/2 and proinflammatory cytokines

**Fig. 1:**
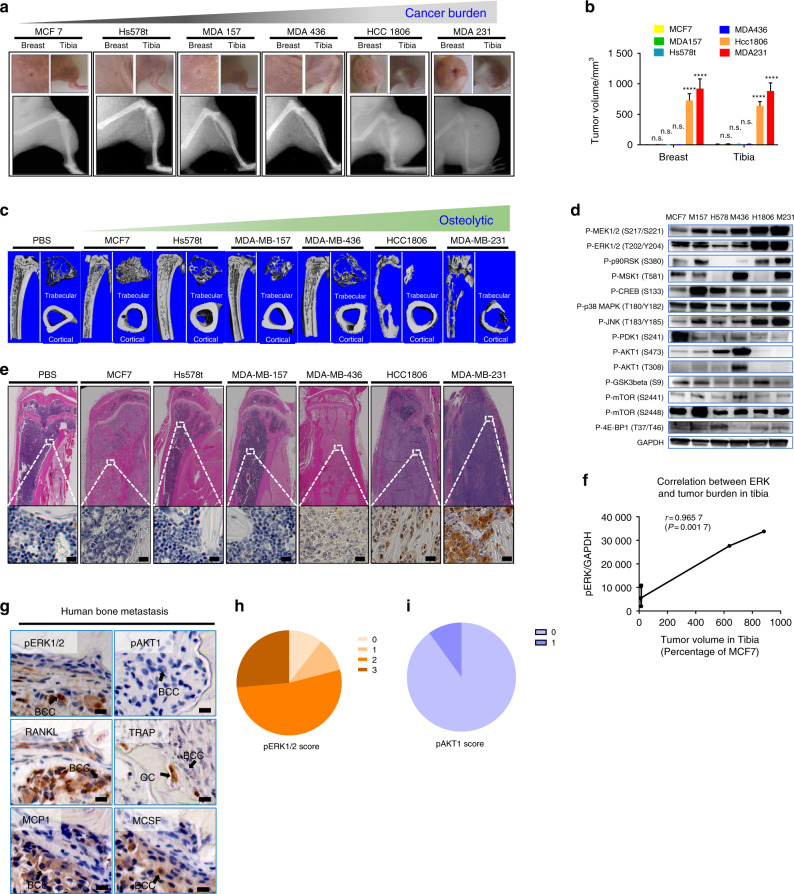
Activation of ERK1/2 by breast cancer cells is correlated with metastatic tumor burden in bone. **a** MDA231 or HCC1806 breast cancer cells displayed more aggressive bone destruction and greater tumor size than MDA436, Hs578t, MDA157 and MCF7 cells (*n* = 3; at 4 weeks following intratibial injection). **b** Tumor growth within murine tibia and breast tissue secondary to breast cancer cell line introduction. Statistical analysis was performed by one-way ANOVA with Dunnett’s multiple comparisons. **c** Micro-CT imaging revealed that breast cancer cells metastatic to bone demonstrate a purely osteolytic phenotype. Imaging was performed in PBS on the HCC1806 and MDA231 groups 4 weeks following intratibial injection; imaging for the MCF7, Hs578t, MDA157, and MDA436 groups was performed at 12 weeks following intratibial injection. **d** Confirmation by Western blot analyses. **e** A representative IHC image showing ERK1/2 activation in BCCs adjacent to an osteolytic lesion. **f** Correlation between ERK1/2 activation and cancer growth in bone. **g** Histologic analysis demonstrated that ERK1/2 activation in BCCs metastatic to bone is associated with increased production of osteoclastogenic cytokines and chemokines. pERK1/2^+^ BCCs are indicated by the presence of arrows. Scale bar: 20 μm. **h** Quantification of pERK1/2^+^ BCCs in human breast cancer bone metastases. Seventeen of 19 (89.5%) lesions positively stained for pERK1/2 in BCCs. Five (26.3%) of these lesions earned scores of 3, indicating strong, complete membrane staining in >10% of cells, 10 (52.6%) earned scores of 2, indicating weak to moderate strength, complete staining in >10% of cells, and 2 (10.5%) lesions garnered a score of 1, indicative of weak and partial staining in >10% of cells, with respect to pERK1/2 microenvironmental signal intensity. **i** Quantification of pAKT1^+^ BCCs in human breast cancer bone metastases. One of 10 (10%) lesions positively stained for pERK1/2 in BCCs, with a score of 1 according to the aforementioned scoring system

In humans, metastatic breast cancers show various degrees of bone destruction. Thus, it is plausible that aggressive, osteolytic BCCs have intrinsically different molecular signatures and signaling from nondestructive BCCs. To simulate the effects of metastatic breast cancer invasion and proliferation in the human skeleton, we injected six different well-characterized human breast cancer cell lines (e.g., MDA-MB-231, HCC1806, MDA-MB-436, MDA-MB-157, Hs578t, and MCF7) (Table S1) into the breasts and tibiae of nude mice. On gross inspection and radiographic analysis at 4 weeks, there was an appreciable difference in growth among the six different cancer cell lines. MDA-MB-231 (MDA231) cells grew the most rapidly, followed by HCC1806 cells. In contrast, MDA-MB-436 (MDA436), MDA-MB-157 (MDA157), Hs578t, and MCF7 cells did not demonstrate remarkable growth patterns (Fig. 1a, b). Similarly, microcomputed tomography (micro-CT) images showed that aggressive cancer cell lines display a different osteolytic phenotype in bone (Fig. 1c). Given that osteoclastogenesis, which is permissive for both tumor survival and aggressive cancer growth in bone, is associated with inflammation, it was postulated that rapidly growing BCCs are inflammatory and demonstrate increased inflammatory kinase signaling.

Kinase screening experiments were performed to compare differential signaling pathway activation between the least (MCF7) and most (MDA231) aggressive breast cancer cell lines. These assays revealed that ERK1/2 activation increased more significantly in MDA231 cells than in MCF7 cells (Fig. S1). Western blotting confirmed that the more aggressive cancer lines MDA231 and HCC1806 expressed higher levels of the active, phosphorylated kinase ERK1/2 (pERK1/2) relative to the nonaggressive cell lines MDA436, MDA157, Hs578t, and MCF7 (Fig. 1d). Furthermore, hematoxylin and eosin staining and immunohistochemistry (IHC) revealed that the increased levels of pERK1/2 in BCCs were associated with an increase in the number of osteolytic bone lesions observed (Fig. 1e). The phosphorylation of extracellular receptor kinase 1/2 (pERK1/2) in BCCs in bone constitutes a distinct molecular change that was correlated with tumor size and the extent of bone destruction observed (Fig. 1c, f). MCF7 cells weakly expressing ERK1/2 grew slower than MDA231 cells highly expressing ERK1/2. MCF7 cell growth in tibiae induced new bone formation (Fig. 1c, e). To identify the inflammatory cytokine profiles of the osteolytic and osteoblastic BCC phenotypes, we analyzed the expression of inflammatory cytokines from MDA231 and MCF7 cells via PCR array. We found that the expression of the osteoclastogenic cytokines IL-1β (89.2-fold) and IL-8 (207.36-fold) was dramatically upregulated in MDA231 cells compared to MCF7 cells (Fig. S2 and Table S2).^25–27^ Trametinib treatment reduced the expression of IL-1β (0.13-fold) and IL-8 (0.03-fold) relative to that of the control MDA231 cell conditions. These results suggest that IL-1β and IL-8 may play a role in the osteolytic phenotype of BCCs with high expression of pERK1/2.

To confirm that upregulation of pERK1/2 expression in breast cancer is associated with osteolytic bone lesions, we analyzed 19 human pathology specimens isolated during surgical interventions for pathologic fractures secondary to metastatic breast cancer. IHC was performed on each sample to determine the expression patterns of pERK1/2, osteoclastogenic and proinflammatory cytokines (RANKL, MCP1, M-CSF, IL-6), TRAP (tartrate-resistant acid phosphatase), and phosphorylated protein kinase B (PKB/AKT1) (Fig. 1g and Table S3). A total of 89.5% of the osteolytic breast cancer metastases to bone expressed pERK1/2 (Fig. 1h), while only 10% of the BCCs within these lesions stained positively for pAKT1 (Fig. 1i). Together, these results suggest that pERK1/2 levels in BCCs may play a role in breast cancer-induced bone destruction.

### pERK1/2-high BCCs promote osteoblast phenotypic conversion and inflammatory mediator secretion

**Fig. 2:**
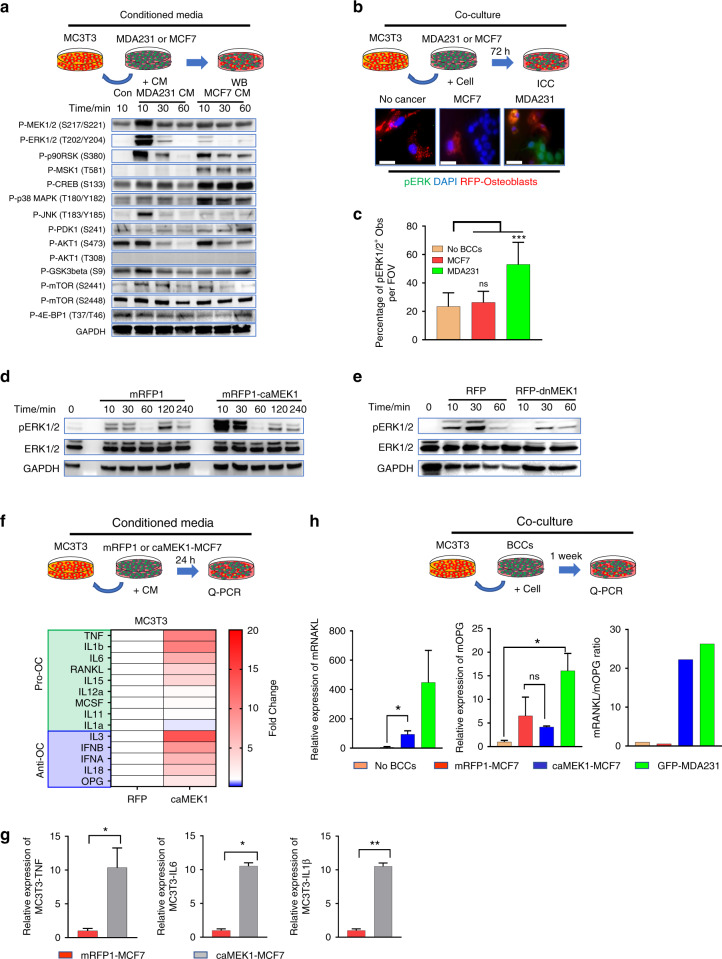
pERK1/2-high BCCs promote the conversion of naïve Obs into Obs expressing osteoclastogenic cytokines. **a** Kinase activation level in MC3T3 cells through conditioned media (CM) derived from cancer cells. Conditioned media (CM) derived from pERK1/2-high MDA231 cells induced ERK1/2 activation in Obs. **b** MDA231 cells promote the conversion of pERK1/2-low MC3T3 cells to pERK1/2-high MC3T3 cells. **c** MDA231 breast cancer cells highly expressing pERK1/2 were observed to histologically promote the conversion of MC3T3 cells weakly expressing pERK1/2 into pERK1/2-high MC3T3 cells under coculture conditions. Statistical analysis was performed by one-way ANOVA with Dunnett’s multiple comparisons. **d** CM derived from caMEK1-MCF7 cells promoted ERK1/2 activation in MC3T3 cells. **e** Lower levels of ERK1/2 activation in MC3T3 cells were observed in the presence of CM derived from dnMEK1-MDA231 cells compared to mRFP1-MDA231-derived CM. **f**, **g** Activation of ERK1/2 in the caMEK1-MCF7-expressing cells promoted the phenotypic conversion of naïve Obs into osteoclastogenic cytokines expressing Obs. *P* values were calculated using Student’s two-tailed *t* test. **h** RANKL/OPG ratios of local Obs increased secondary to interactions with caMEK1-MCF7-expressive BCCs. *P* values were calculated using the two-tailed *t*-test

Breast cancer metastases have a disproportionate predilection for bone. Clarification of the mechanisms by which breast cancer can adapt to and expand within the structurally rigid, calcified matrices of bone is needed. Osteoblasts help maintain homeostasis within the bone microenvironment in myriad ways. These cells have been directly implicated in the process of osteolysis induced by metastatic BCCs.^28,29^ We hypothesized that aggressive osteolytic BCCs convert normal osteoblasts into tumor-associated osteoblasts (TAObs) by altering the kinase activation of osteoblasts. TAObs secrete inflammatory osteoclastogenic cytokines and growth factors that promote bone resorption and inhibit bone formation, thereby altering the bone microenvironment to favor tumor survival and infiltration of resident bone. MC3T3-E1 (MC3T3) premature osteoprogenitor cells, a well-characterized and commonly used preosteoblast derived from mouse calvariae, were treated with conditioned media from pERK1/2-low osteoblastic MCF7 cells or pERK1/2-high osteolytic MDA231 cells, and kinase activity was subsequently measured. Conditioned media isolated from MDA231 cells dramatically induced ERK1/2 activation in MC3T3 cells, while conditioned media from MCF7 cells did not dramatically increase ERK1/2 activation (Fig. 2a). Similarly, when osteoblasts were cocultured with pERK1/2-low MCF-7 cells, they still expressed low levels of pERK1/2. In contrast, the coculture of MC3T3 cells with pERK1/2-high MDA-231 cells induced ERK1/2 activation (Fig. 2b, c). These observations suggest that interactions between osteolytic BCCs with high expression of pERK1/2 and host osteoblasts at the bone-metastatic cancer interface may promote inflammatory kinase activation in osteoblasts compared to osteoblastic BCCs with low expression of pERK1/2.

To test whether ERK1/2 activation in BCCs is critical for the conversion of normal osteoblasts to aggressive pERK1/2-high osteoblasts, we generated two cell lines. The first cell line was pERK1/2-low MCF7 cells with a constitutively active MEK1 mutation (caMEK1-MCF7) (Fig. S3a). The second cell line was pERK1/2-high MDA231 cells that expressed a dominant-negative MEK1 mutation (dnMEK1-MDA231) (Fig. S3b). Treatment of osteoblasts with conditioned media isolated from the caMEK1-MCF7 cells significantly induced ERK1/2 activation compared to that of the controls (Fig. 2d). Treatment of osteoblasts with conditioned media isolated from the dnMEK1-MDA231 cells yielded reduced levels of ERK1/2 activation compared to that of the mRFP1-MDA231 group (Fig. 2e). Quantitative PCR (q-PCR) analysis showed that constitutively active MEK1 expression modified the inflammatory cytokine profile of pERK1/2-low MCF7 cells (Fig. S3c). Expression of the osteoclastogenic cytokines IL-1β and IL-8 was significantly upregulated in the caMEK1-MCF7 cells (Fig. S3d). q-PCR analysis revealed significant alterations in inflammatory cytokine expression by MC3T3 cells exposed to caMEK1-MCF7-derived conditioned media (Fig. 2f). Production of the osteoclastogenic cytokines TNF, IL-6, and IL-1β was significantly increased 24 h after exposure to osteoblasts treated with conditioned media derived from the caMEK1-MCF7 cells compared to the control conditioned media (Fig. 2g). Moreover, coculturing MC3T3 cells with the caMEK1-MCF7 cells successfully induced the conversion of RANKL/OPG low osteoblasts to RANKL/OPG high osteoblasts (Fig. 2h). A dominant-negative MEK1 mutant osteoblast cell line (dnMEK1-MC3T3) and constitutively active MEK1 mutant osteoblast cell line (caMEK1-MC3T3) (Fig. S3e) were constructed to evaluate the role of ERK signaling in interactions between these cell types. To confirm the critical role of ERK1/2 activation in the inflammatory phenotypic conversion of osteoblasts, we examined the expression of osteoclastogenic cytokines and osteogenic genes in the caMEK1-MC3T3 cells. The expression of numerous inflammatory cytokines increased with activation of the ERK signaling cascade (Fig. S3f). Osteoblastic maturation-related gene expression was significantly reduced in the caMEK1-MC3T3 cells compared to the control MC3T3 cells (Fig. S3g). These findings suggest that osteolytic BCCs with high expression of pERK1/2 may promote bone destruction by increasing osteoclastogenesis and by inhibiting bone formation via ERK1/2 activation in osteoblasts.

### Aggressive osteolytic BCCs are associated with an increase in the population of osteoblasts highly expressing pERK1/2

**Fig. 3:**
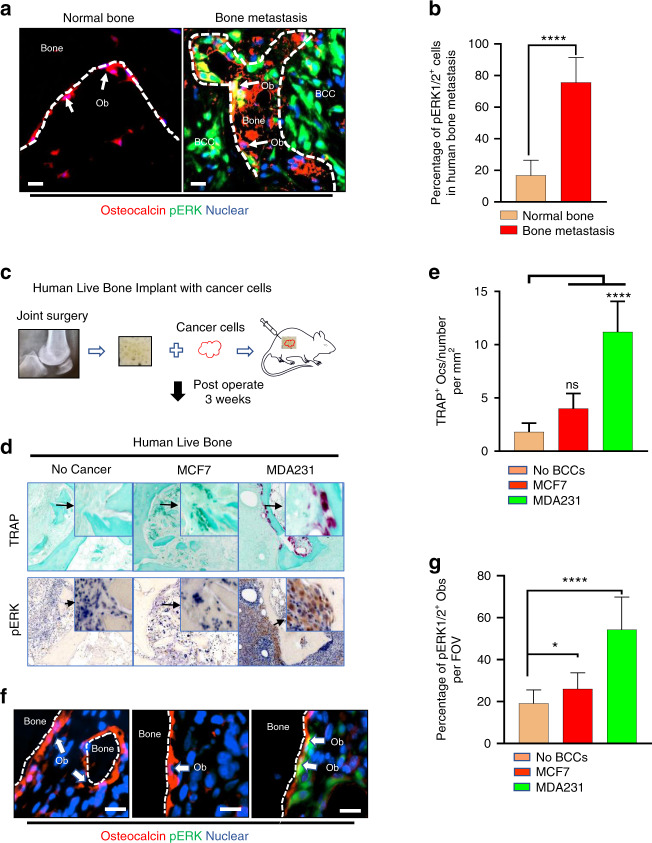
Aggressive osteolytic BCCs are associated with increased pERK1/2-high Obs. **a** Multiplex immunofluorescence labeling of Obs (osteocalcin) and pERK1/2 in the area surrounding BCCs. Two subpopulations of Obs (arrows), with (yellow) and without (red) active ERK1/2, coexist surrounding metastatic BCC lesions (encircled by dotted lines). Scale bar: 20 μm. **b** Comparison of pERK1/2 IHC positivity scores in Obs isolated in the setting of human breast cancer bone metastases (*n* = 5) and normal bone (*n* = 9). *P* values were calculated using a two-tailed Student’s *t* test. **c** Schematic representation of the experimental design mimicking human breast cancer bone metastasis using human bone biopsy implantation in a murine model. **d** Representative images showing bone implants under different experimental conditions. High magnification shows pERK1/2 in Obs. **e** Quantification of TRAP ^+^ osteoclast number under different experimental conditions. TRAP ^+^ osteoclast number calculated from images from the experiment in (**d**). Statistical analysis was performed by one-way ANOVA with Dunnett’s multiple comparisons. **f** Histologic images of implanted BCC bone constructs under different experimental conditions. High magnification shows pERK1/2 in Obs. Scale bar: 20 μm. **g** Quantification of pERK1/2^+^ Obs in human bone implanted with breast cancer cells. pERK1/2^+^ Obs number calculated from images from the experiment in (**f**). Statistical analysis was performed by one-way ANOVA with Dunnett’s multiple comparisons

For analysis of whether breast cancer bone metastasis was associated with osteoblastic ERK1/2 activation, three samples from human breast cancer metastasis to bone and uncompromised bone samples were surgically resected during tumor debulking/resection and total knee arthroplasty (TKA), respectively, and stained for pERK1/2 and osteocalcin (Fig. 3a). ERK1/2 activation was significantly elevated in a subpopulation of osteoblasts from these human breast cancer bone metastasis specimens relative to the levels observed in those taken from patients who underwent TKA (Fig. 3b).

To test whether BCCs highly expressing pERK1/2 induce osteoclastogenesis in human bone, we generated a mouse model mimicking the human bone microenvironment using implanted human cells (Fig. 3c). Similar to findings obtained from mouse intratibial injection models, MDA231 cells significantly induced osteoclastogenesis to a greater degree than MCF7 cells in live cancer-bearing bones (Fig. 3d, e). To confirm ERK1/2 activation in osteoblasts by pERK1/2-high BCCs, we performed IHC for pERK1/2 and osteocalcin (Fig. 3f). Consistent with observations of human breast cancer bone metastasis samples, the presence of MDA231 cells highly expressing pERK1/2 increased the number of pERK1/2-high osteoblasts at the bone-breast cancer interface (Fig. 3g). These results suggest that BCCs highly expressing pERK1/2 may convert quiescent osteoblasts into osteoclastogenic cytokine-secreting osteoblasts (oObs) with high expression of pERK1/2.

### MEK1/ERK1/2-mediated inflammatory interactions accelerate bone destruction and cancer growth in vivo

**Fig. 4:**
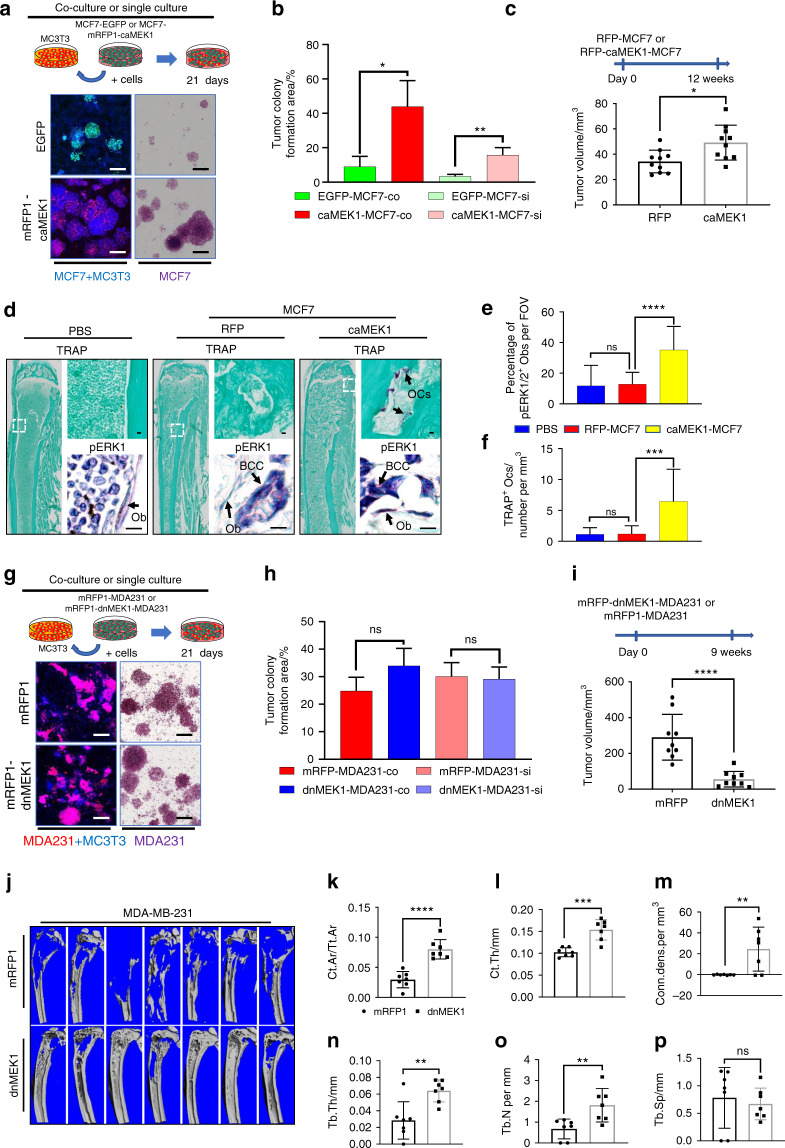
MEK1/ERK1/2-mediated inflammatory interactions between aggressive breast cancer cells and osteoblasts accelerate bone destruction and cancer growth. **a** ERK1/2 activation in MCF7 cell growth under both single culture conditions and coculture conditions. **b** BCC colony formation was quantified by colony formation area. The mean colony area size was computed from images from the experiment in (**a**). Scale bar, 200 μm. **c** caMEK1 expression in MCF7 cells in bone destruction and cancer growth. *n* = 10 mice per group. **d** pERK1/2 immunostaining and TRAP staining of tibiae harvested from the mRFP-MCF7- or mRFP-caMEK1-MCF7-exposed mice. Scale bar: 100 μm. **e** Quantification of TRAP ^+^ osteoclast number under different experimental conditions. TRAP ^+^ osteoclast number calculated from images from the experiment in (**d**). **f** Quantification of pERK1/2^+^ osteoblasts in murine tibiae with breast cancer cells. pERK1/2^+^ osteoblast number calculated from images from the experiment in (**d**). **g** dnMEK1 expression in MDA231 cells grown under both single culture conditions and coculture conditions. **h** BCC colony formation was quantified by the colony formation area. The mean colony area size was computed from images from the experiment in (**g**). Scale bar, 200 μm. **i** dnMEK1 expression in MDA231 cells in bone destruction and cancer growth. *n* = 9 mice per group. **j** Micro-CT images of tibiae harvested from nonmetastatic tumor-bearing and tumor-bearing mice 9 weeks after injection. Cortical area fraction Ct.Ar/Tt. Ar (**k**), cortical thickness (**l**), connectivity density (**m**), trabecular thickness (**n**), trabecular number (**o**), and trabecular separation (**p**) were determined using a Scanco μCT 35 system. *n* = 7 mice per group. *P* values were calculated using the two-tailed *t*-test in Fig. 4b, c, h, I, k, l, m, n, o, and p. One-way ANOVA was performed with Dunnett’s multiple comparisons in Fig. 4e and f

The ERK1/2 pathway is known to regulate cell proliferation.^30^ We found that the caMEK1-MCF7 cells had stronger growth than the vector-transfected MCF7 cells under both single-culture and coculture conditions with MC3T3 cells (Fig. 4a, b). To test whether ERK1/2 activation in BCCs critically regulates cancer burden in bone, we examined the growth of the caMEK1-MCF7 cells in the mouse tibiae. Increased cancer growth was observed in the caMEK1-MCF7 group relative to the control group (Fig. 4c). A large increase in the pERK1/2^+^ osteoblast population was observed at the bone-caMEK1-MCF7 cell interface (Fig. 4d, e), and these cells subsequently induced higher levels of bone destruction (Fig. 4d, f). Conversely, dnMEK1 expression did not affect MDA231 cell growth under in vitro single culture and coculture conditions (Fig. 4g, h). Western blot results revealed lower levels of ERK1/2 activation by ~70% in the dnMEK1 mutant cells compared to the controls (Fig. S3b). However, it is possible that levels of ERK1/2 activation as low as 30% may be sufficient for MDA231 cell proliferation in vitro. The growth of the dnMEK1-MDA231 cells was also examined using murine tibial injection models to determine whether dnMEK1 expression can reduce MDA231 growth in vivo. Interestingly, dnMEK1-MDA231 cell growth was dramatically inhibited in bones in vivo, in contrast our observations under in vitro conditions (Fig. 4i). To investigate whether BCC-induced osteolysis is reduced by ERK1/2 inhibition, we analyzed mouse tibiae by micro-CT. The destruction of bone by BCCs was dramatically reduced upon exposure to the dnMEK1-MDA23 cell line compared to the control MDA231 cell line (Fig. 4j-p). This finding suggests that the growth of BCCs highly expressing pERK1/2 is dependent on the presence of tumor-associated stromal cells. The interaction of native osteoblasts with phenotypically aggressive BCCs highly expressing pERK1/2 generates osteoblasts highly expressing pERK1/2 that not only enhance osteoclastogenesis but also further promote BCC growth within bone. To test whether pERK1/2^+^ osteoblasts promote BCC growth, we cocultured GFP-MDA231 cells with the dnMEK1-MC3T3 or caMEK1-MC3T3 cells (Fig. S4a). Although the growth of BCCs highly expressing pERK1/2 was less inhibited when these cells were cocultured with the dnMEK1-MC3T3 cells, it was significantly increased under the caMEK1-MC3T3 coculture condition (Fig. S4b).

To identify which MC3T3-derived molecules are important for BCC growth, we analyzed the expression of MC3T3 genes using species-specific primers (Table S4). The expression of numerous inflammatory cytokines, chemokines, and growth factors was upregulated in the caMEK1-MC3T3 cells (Fig. S4a) and downregulated in the dnMEK1-MC3T3 (Fig. S4b) cells relative to the controls. BMP5 and GDF10 (BMP3b) expression was particularly altered by changes in ERK1/2 activation, although the functions of these genes in breast and metastatic cancers have not been well characterized. These results suggest that ERK1/2-mediated positive feedback between BCCs and osteoblasts may both accelerate cancer growth and induce osteoclastogenesis. ERK1/2 targeting therapy may thus regulate the activity of osteolytic BCCs highly expressing pERK1/2 by blocking this positive feedback between BCCs and native bone resident cells.

### ERK1/2 inhibition restores osteoblastic differentiation and reduces inflammatory cytokine production in breast cancer-preosteoblast cocultures

**Fig. 5:**
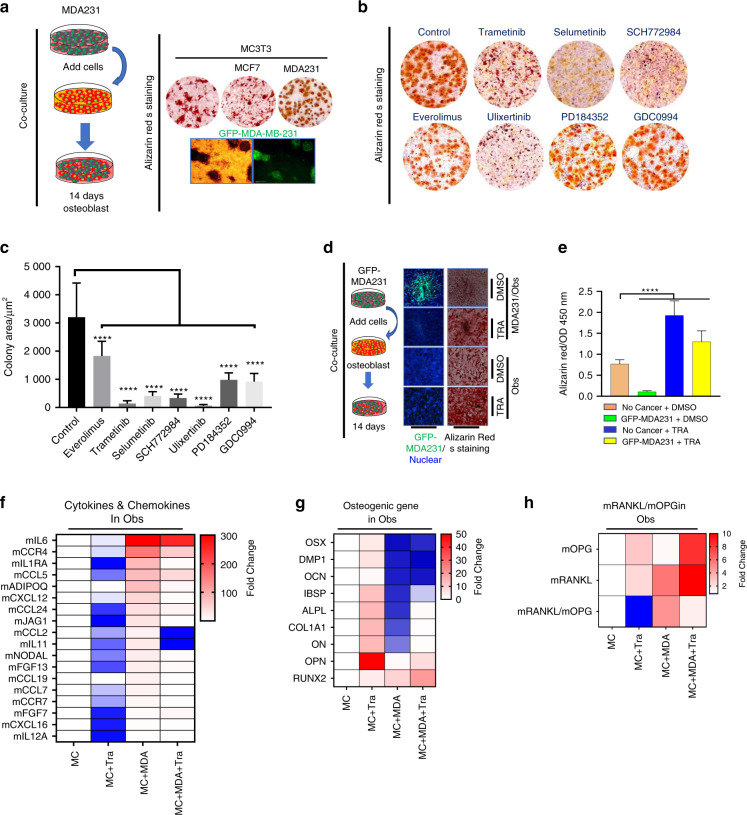
Pharmacologic inhibition of ERK1/2 induces bone formation and reduces osteoclastogenic cytokine expression in osteoblast-associated BCCs. **a** Clinically aggressive BCCs highly expressive of pERK1/2 inhibit preosteoblast maturation. The MDA-MB-231 cells are labeled with GFP. **b** Pharmacologic inhibition of ERK1/2 phosphorylation restores osteoblastic mineralization during differentiation from osteoblast precursor cells; both processes are impaired in the presence of highly pERK1/2-expressing BCCs. Alizarin Red S staining and quantification of cancer cells 14 days after treatment with dimethyl sulfoxide (DMSO) or the indicated inhibitors. MEKi; trametinib 40 nmol·L^−1^ (GSK1120212), selumetinib 300 nmol·L^−1^ (AZD6244) and PD184352 300 nmol·L^−1^, ERKi; ulixertinib 600 nmol·L^−1^, SCH772984 100 nmol·L^−1^ and GDC-0994 40 nmol·L^−1^, mTORC1i; everolimus 40 nmol·L^−1^ (RAD001). **c** Quantification of the mean tumor colony area based on the pharmacologic pERK1/2 inhibition experiment in (**b**). Statistical analysis by one-way ANOVA with Dunnett’s multiple comparisons. **d** Osteogenic differentiation was determined using Alizarin Red S staining, which revealed that trametinib not only inhibited BCC growth but also induced bone formation. MDA-MB-231 cells were labeled with GFP for visualization. **e** Alizarin Red S staining quantification in images from the experiment in (**d**). Statistical analysis was performed by one-way ANOVA with Dunnett’s multiple comparisons. **f** Differential heat map of inflammatory cytokine, chemokine, and growth factor expression in osteoblasts cocultured with MDA231 cells in the presence of trametinib. **g** Differential heat map of osteogenic gene expression in osteoblasts cocultured with MDA231 cells in the presence of trametinib. **h** Differential heat map of RANKL/OPG ratio changes among osteoblasts treated with trametinib

MC3T3 cells undergo a series of differentiation stages with increasing mineralization in vitro. MDA231 and MCF7 BCCs were cocultured with early-stage predifferentiated MC3T3-E1 osteoblasts. MDA231 cells grew more vigorously than MCF7 cells in these cocultures. In addition, osteoblast mineralization was inhibited to a greater extent in the MDA231 cocultures than in the MCF7 cocultures (Fig. 5a). The growth of BCCs cocultured with MC3T3 cells in the presence and absence of ERK1/2 phosphorylation inhibitors was subsequently examined (Fig. 5b). The majority of the MEK inhibitors evaluated dramatically reduced cancer growth (Fig. 5c). Trametinib was chosen from the six commercially available inhibitors of ERK1/2 phosphorylation because it demonstrated the greatest pERK1/2 inhibition in BCCs. Moreover, inhibition of ERK1/2 phosphorylation not only reduced BCC growth but also restored mineralization and osteoblastic differentiation of MC3T3 cells under coculture with BCCs. This enhanced mineralization of osteoblasts was also observed with trametinib treatment in single culture conditions compared to the control, verifying its pERK1/2 inhibitory effects (Fig. 5d, e). Gene expression profile analyses demonstrated that trametinib not only reduced the expression of inflammatory cytokines and chemokines in vitro (Fig. 5f) but also normalized and restored the expression of osteogenic genes such as integrin binding sialoprotein, collagenase I, osteonectin, and alkaline phosphatase (Fig. 5g). RANKL and OPG expression was measured by q-PCR in osteoblasts cocultured with MDA231, and a steep increase in the RANKL/OPG ratio was observed when osteoblasts were cocultured with aggressive BCC lines. However, coculturing osteoblasts with the same BCC lines and trametinib successfully normalized the RANKL/OPG ratio (Fig. 5h). Together, these results suggest that ERK1/2 activation in osteoblasts inhibits mineralization and promotes osteoclastogenic cytokine expression. Moreover, MEK inhibitors may protect bone by restoring skeletal homeostasis.

### pERK1/2 targeting reduces cancer-induced bone destruction in vivo

**Fig. 6:**
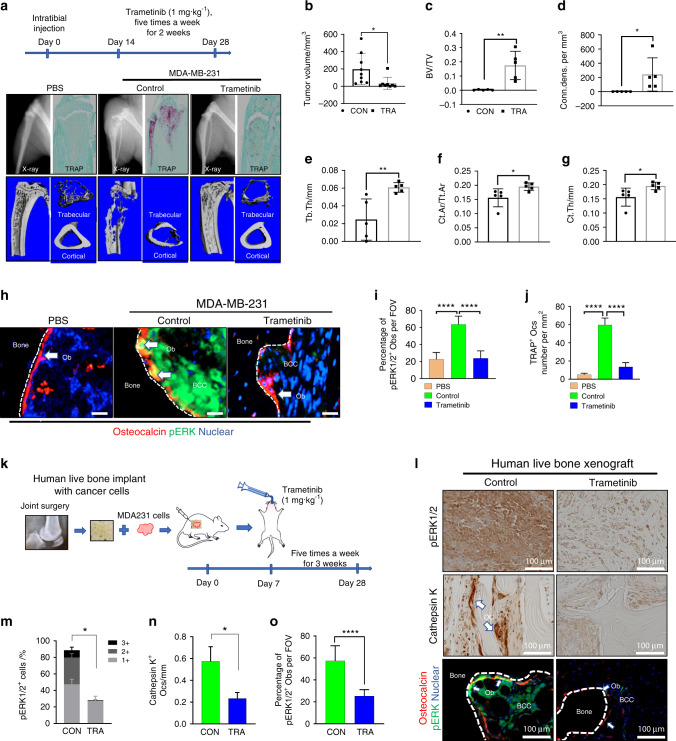
Pharmacologic targeting of the MEK1/2-pERK1/2 axis decreases cancer-induced bone destruction. **a** MDA-MB-231 cells were injected in the tibiae of Balb/c nude mice. Treatments began on day 14 after injection. Trametinib (1 mg/kg oral five times weekly) was administered by oral gavage (o.g.) for 2 weeks. Representative micro-CT images of tibiae collected from nontumor-bearing and tumor-bearing mice after trametinib treatment. **b** Comparison of tumor volumes (in mm^3^) in murine tibiae (control: *n* = 9; trametinib: *n* = 8). *P* values were calculated using a two-tailed Student’s *t* test. Bone volume fraction (**c**), connectivity density (**d**), trabecular thickness (**e**), cortical area fraction (**f**), and cortical thickness (**g**) determined using a Scanco μCT 35 system. *n* = 5 mice per group. *P* values were calculated using the two-tailed *t*-test. **h** Representative images of pERK1/2^+^ Obs found in the trametinib-treated and untreated murine tibiae. **i** Quantification of pERK1/2^+^ osteoblasts in murine tibiae with breast cancer cells. pERK1/2^+^ Obs number calculated from images from the experiment in (**h**). One-way ANOVA was performed with Dunnett’s multiple comparisons. **j** Quantification of TRAP ^+^ osteoclast number under different experimental conditions. TRAP ^+^ osteoclast number calculated from images from the experiment in (**a**). One-way ANOVA was performed with Dunnett’s multiple comparisons. **k** Schematic representation of the trametinib experimental design using live human bone samples. **l** A representative image showing ERK1/2 activation in the bone microenvironment, which includes osteoblasts highly expressing pERK1/2 and Cathepsin K ^+^ osteoclasts surrounding MDA231 cells. Scale bar: 100 μm. Quantification of pERK1/2^+^ cells (**m**), Cathepsin K ^+^ osteoclasts (**n**), and pERK1/2^+^ Obs (**o**) in (**i**) (control: *n* = 6; trametinib: *n* = 9). Two-tailed Student’s *t* tests were performed

Translational investigation of the aforementioned in vitro findings was pursued to determine the extent to which available pERK1/2 inhibitors protect tumor-laden bones from osteolysis in living organisms. MDA231 breast cancer-bearing mice were randomized to receive 1 mg·kg^−1^ trametinib via oral gavage or placebo. X-ray, micro-CT, and TRAP activity analyses showed that trametinib treatment dramatically protected host bone from breast cancer-induced destruction (Fig. 6a). Treatment with trametinib significantly suppressed the cancer burden in bone (Fig. 6b). Micro-CT results showed that trametinib reduced BCC-induced bone destruction relative to the placebo group (Fig. 6c-g). pERK1/2 expression was examined in osteoblasts juxtaposed to aggressive MDA231 cells with high expression of pERK1/2 in mouse tibiae (Fig. 6h). Trametinib reduced the population size of osteoblasts with high expression of pERK1/2 in the tumor-associated bone microenvironments of affected tibiae (Fig. 6i). A decrease in the number of TRAP-positive osteoclasts present in the tibiae of mice was observed with trametinib treatment in addition to a decrease in the pERK1/2^+^ osteoblast population (Fig. 6j). Trametinib is known to inhibit the growth of cancer cells highly expressing pERK1/2. To investigate whether trametinib not only inhibits BCC growth but also reduces the osteoclastogenic ability of BCCs, we analyzed osteoclastogenic gene expression in both BCCs using human-specific primers and bone cells using mouse-specific primers. Gene expression profile analyses demonstrated that trametinib reduced the expression of inflammatory cytokines such as IL-1α, IL-1β, and IL-23α in both BCCs (Fig. S5a) and bone cells (Fig. S5b). These results suggest that targeted ERK1/2 therapy inhibits BCC-induced bone loss and BCC growth in the BCC-bearing skeletal compartment.

To test whether trametinib directly inhibits osteoclastogenesis, we performed an in vitro RANKL-induced osteoclastogenesis assay using the mouse macrophage-like cell line RAW264.7 (Raw). MC3T3-derived CM (MC3cm) was pretreated with or without MDA231-derived CM (MDAcm) and applied to Raw cells. MDAcm^−^/MC3cm^+^ induced Raw cell proliferation but reduced osteoclast formation. Trametinib treatment rescued osteoclast formation under the same experimental conditions (Fig. S6b). MDAcm^+^/MC3cm^+^ and MDAcm^+^/MC3cm^−^ treatment also induced Raw cell proliferation but inhibited osteoclast formation (Fig. S6c). Based on these results, we hypothesized that ERK activation may induce osteoclast precursor cell proliferation but inhibit the differentiation of osteoclast precursor cells into osteoclasts.

We performed RANKL-induced osteoclastogenesis assays under both high and low cell concentration seeding conditions. Osteoclast populations were significantly reduced under the high-concentration seeding condition compared to the low-concentration seeding condition. Interestingly, trametinib dramatically inhibited osteoclast formation under low-concentration seeding conditions but to a lesser extent under high-concentration seeding conditions (Fig. S6d). Similarly, Hotokezaka et al. showed that the effect of MEK inhibitors on osteoclastogenesis varied according to the initial cell culture density during culture.^31^ These results suggest that activation of ERK1/2 induces osteoclast precursor cell proliferation but inhibits differentiation to osteoclasts.

A multitude of differences exist between the bone microenvironments of mice and humans. Our model lacks T and B cells, which are important in the regulation of cancer development and metastasis. We instead sought to mimic the human bone microenvironment in a mouse model using live human bone to extrapolate the in vitro and in vivo murine findings described herein to humans and validate the clinical efficacy of MEK-targeting therapy (Fig. 6k). The effect of trametinib was examined in a model of human breast cancer bone metastasis. Histological imaging (Fig. 6l) showed that treatment with trametinib inhibited the growth of clinically aggressive BCCs highly expressing pERK1/2 (Fig. 6m), decreased osteoclast formation (Fig. 6n), and inhibited the formation of human osteoblasts highly expressing pERK1/2 (Fig. 6o). Overall, trametinib efficaciously modulated the human bone microenvironment for the clinical management of aggressive osteolytic breast cancer metastases.

## Discussion

Metastasis is the principal cause of death among breast cancer patients. The osteotropism of breast cancer distinguishes it as one of the most common primary tumor types associated with metastatic bone disease. Local and distant tumor growth is influenced by interactions between cancer cells and the tumor microenvironment, which involves infiltrating tumor-associated nontumor cells, extracellular matrix proteins, and secretory molecules.^32^ As calcified tissue, the growth of cancer cells within bone is difficult in the absence of osteoclastogenesis and bone resorption. However, osteoclastogenesis is not critical for osteoblastic cancer proliferation, such as in the case of prostate cancer.^33^ Osteoclast inhibitors such as bisphosphonates and denosumab are commonly prescribed for patients with breast cancer metastases to bone.^24,34^ Although bisphosphonates have been shown to reduce the number of skeletal metastases that develop, they do not consistently prevent pathologic fracture.^35,36^ Denosumab, a monoclonal antibody targeting RANKL, is plagued by the same issues, in addition to being less cost-effective. Both bisphosphonates and denosumab are associated with avascular necrosis and the acquisition of atypical fractures.^37–39^ These therapies primarily focus on the inhibition of osteoclast formation and activity, although cancer-induced bone resorption proceeds by multiple inherently complex and dynamic processes. Given the persistent inadequacies of existing medication regimens, as well as the substantial clinical sequelae of skeletal metastases, there is a dire need for more effective therapeutic options for advanced osteolytic metastatic breast cancers. We demonstrate that osteolytic BCCs can induce phenotypic conversion of osteoblasts to promote neoplastic growth in bone. These results suggest that osteoblasts can also be a therapeutic target for breast cancer metastases to bone.

Protein kinases play a key role in oncogenic signaling and constitute a major focus in the development of targeted cancer therapies.^40,41^ The orderly regulation of inflammation by kinase activation is important for skeletal homeostasis. Abnormal kinase activation underlies the acute inflammatory responses evoked by metastatic BCCs in the destruction of bone. Our kinase screening assay of known BCC lines highlighted the importance of ERK1/2 in these inflammatory signaling cascades. The Raf/MEK/ERK signaling pathway is vital to the maintenance of normal human physiology^42^ and is commonly dysregulated in several human cancers.^43^ MEK inhibitors effectively impede the growth of a variety of human cancer cell lines. Our previous report showed that osteoclastogenesis was promoted by ERK1/2 activation of osteoblasts in a TiO_2_ nanoparticle-induced bone resorption model.^44^ Here, we showed that pERK1/2^+^ BCCs and pERK1/2^+^ osteoblasts collaborate to promote osteolysis by creating an inflammatory microenvironment within bone. ERK1/2 activation is important for cellular proliferation. However, if osteolytic breast cancer fails to induce bone resorption, cancer cells remain trapped within the bone marrow. We demonstrate that activation of ERK1/2 in aggressive osteolytic BCCs is important for both the proliferation of cancer cells and cancer cell-induced bone resorption. Osteoclastogenesis–enhanced through the collaboration of cancer cells and osteoblasts—crucially promotes the growth of cancer cells in bone. Although the expression of dnMEK1-MDA231 did not significantly reduce BCC proliferation in vitro (Fig. 4g, h), the growth of these cells was dramatically reduced in vivo (Fig. 4i). Bone resorption was also dramatically inhibited by dnMEK1 expression in MDA231 cells (Fig. 4j-p). caMEK1-MCF7 cell growth significantly increased both in vitro and in vivo, which was associated with a significant increase in local osteoclastogenesis (Fig. 4f) and a mild increase in cancer cell volume in vivo (Fig. 4c). Furthermore, the caMEK1-MCF7 cells resulted in the expression of a higher concentration of osteoclastogenic cytokines by local osteoblasts (Fig. 2f, g, and h). This upregulated set of osteoclastogenic cytokines includes IL1-β and IL-8 (Fig. S3d). IL-1β and IL-8 were highly expressed in MDA231 cells compared to MCF7 cells; trametinib administration reduced the expression of both IL-1β and IL-8 by MDA231 cells (Fig. S2). TNF expression was also found to increase in MDA231 cells, although to a lesser extent relative to IL-1β and IL-8. This finding is significant due to the noteworthy role TNF plays in inflammatory osteolysis both directly by promoting osteoclast differentiation and RANK upregulation via the NF-κB pathway.^45^ These results demonstrate that pERK1/2^+^ BCCs may induce osteoclastogenesis through the activation of ERK1/2 in osteoblasts in response to locally upregulated IL-1β and IL-8.

To determine whether ERK1/2 activation is required for the pathogenesis of osteolytic breast cancer, we used a RAS/RAF mutant MDA-MB-231 breast cancer cell line. RAS or RAF mutations occur in 47% of metastatic melanoma cases,^46^ and KRAS mutations are found in ~25%–27% of human lung adenocarcinomas.^47^ RAS, RAF, and MEK mutations, however, are rarely observed—at rates of ~1%–6%—in breast cancer.^48^ Thus, it is plausible that in breast cancers, ERK activation may proceed via persistent upstream signaling rather than constitutively active RAS, RAF, or MEK mutants. Growth factors coupled to tyrosine kinase-containing membrane receptors (e.g., EGF, IGF-1, prolactin, the heregulin family, insulin, and TGF-α and TGF-β) serve as major regulators of ERK.^49^ Our results show that pERK1/2^+^ osteoblasts can induce BCC growth, enabling these BCCs to adapt to the bone microenvironment through the release of inflammatory cytokines and growth factors (Fig. S4). It remains to be determined which event—ERK1/2 activation in osteoblasts or BCCs—precedes and begets the other. Given the ability of MEK1 inhibitor therapy to prevent inflammatory interactions between osteoblasts and BCCs, our results support the use of MEK1 inhibitor therapy to reduce the osteolysis induced by breast cancer metastases to bone.

Trametinib is a selective and highly potent small molecule inhibitor of MEK1/2 with high oral bioavailability currently approved by the FDA for the treatment of unresectable or metastatic melanoma with BRAF V600E or V600K mutations. The median progression-free survival of metastatic melanoma patients on trametinib therapy was 4.8 months compared to 1.5 months in the chemotherapy group (dacarbazine or paclitaxel) (95% CI: 4.3, 4.9).^50^ Currently, there is a lack of FDA-approved agents for patients with metastatic breast cancer. Our data show that trametinib (1 mg·kg^−1^) monotherapy affects osteolytic BCCs by hindering the development of inflammatory osteolytic conditions in the skeletal microenvironment (Fig. 6). We posit that ERK1/2 targeted therapy may be a new class of alternative adjuvant therapy for patients with osteolytic breast cancer highly expressing pERK1/2 in a precision medicine paradigm by altering the bone microenvironment, even under conditions of acquired drug resistance by cancer cells. Existing standard-of-care treatments for breast cancer bone metastases have several side effects on bone. Chemotherapy and radiotherapy enhance bone damage and inhibit bone remodeling^51^ by inducing osteocyte death and osteoclastogenesis through increased production of the inflammatory cytokines IL-6 and IL-11.^52^ This inflammatory cytokine release is regulated by ERK1/2.^53^ Inhibition of ERK1/2 signaling may provide a novel strategy to reduce bone damage induced by chemotherapy and/or radiotherapy in the setting of metastatic breast cancer.

In humans, melanoma-associated fibroblasts inhibit the proliferation and function of melanoma-specific cytotoxic T cells through IL-1.^54^ Previous studies have shown that combined MEK inhibition and antibody-mediated PD-1/PD-L1 blockade sensitizes RAS/RAF-mutant cancer cells to apoptosis.^55,56^ Our study clearly demonstrates that ERK1/2 activation induces IL-1β expression in BCCs and osteoblasts. Overall immune system modulation by BCCs was not investigated due to the use of immunodeficient mice across all experiments. Nonetheless, many osteoclastogenic cytokines have anticytotoxic T cell abilities.^57^ Therefore, inhibiting the expression of these cytokines using MEK inhibitors may increase the efficacy of existing immune checkpoint therapies. We tested the therapeutic effect of trametinib in an experimental model that mimics human bone metastasis using live human bone and breast cancer cell lines. Although we did not observe the anticancer effects of human immune cells in bone, modification of our existing model would afford ready evaluation of immune checkpoint therapy for cancers metastatic to bone.

In summary, we provided evidence that the ERK1/2-mediated interaction between BCCs and osteoblasts promotes bone destruction. [1] ERK1/2 is activated in specimens derived from patients with osteolytic breast cancer and in aggressive human osteolytic breast cancer cell lines. [2] Osteolytic BCCs promote ERK1/2 activation in osteoblasts. [3] ERK1/2-activated BCCs and osteoblasts express greater levels of osteoclastogenic cytokines than ERK1/2-inactivated controls. [4] Osteoblastic MCF7 cells are converted to osteoblastic/osteolytic MCF7 cells by ERK1/2 activation. [5] Osteolytic bone destruction by osteolytic MDA231 cells was reduced through ERK1/2 inhibition, even though dnMEK1-MDA231 cell proliferation did not significantly change in vitro. [6] Trametinib reduced osteoclastogenic cytokine expression in tumor and bone cells in vitro and in vivo.

In addition to supporting the use of targeted ERK1/2 therapy to hinder cancer cell proliferation, we suggest several therapeutic benefits of ERK1/2 inhibition in aggressive osteolytic BCCs compared to bisphosphonate and RANKL-antibody treatment: [1] suppression of osteolytic breast cancer proliferation by inhibiting the production of protumor molecules from host bone and osteoblasts and [2] amelioration of osteolysis by reducing rates of bone resorption and promoting new bone formation. ERK1/2-activated BCCs incite osteolytic bone destruction. However, even in the acquisition of drug resistance by BCCs, trametinib persists in its ability to inhibit ERK1/2 in bone cells. By restoring homeostasis within the bone microenvironment, ERK1/2-centered treatment modalities will enhance clinical outcomes for patients with advanced osteolytic metastatic breast cancer and reduce the incidence of pathologic fractures, cancer-related pain severity, and consequent disability. These findings firmly establish a rationale for the application of MEK/ERK inhibitors in the treatment of osteolytic breast cancer.

## Materials & methods

### Animals

BALB/c nude mice were purchased from Charles River Laboratories. Mice were housed in a pathogen-free environment maintained by the Institute of Comparative Medicine. All mice were maintained according to the Guide for the Care and Use of Laboratory Animals published by the National Institutes of Health (NIH). The mice were provided sterilized food and water and housed in negative pressure isolators with 12 h light/dark cycles.

### Antibodies

Anti-pERK1/2 (Trh202/Ty4204) (cat. 9102S), anti-pAKT1 (Ser473) (cat. 4060S), anti-pAKT1 (Thr308) (cat. 13038S), anti-pEK1/2 (Ser217/Ser221) (cat. 9154S), anti-pMSK1 (Thr581) (cat. 9595S), anti-p-p90RSK (Ser380) (cat. 11989S), anti-mTOR (Ser2448) (cat. 5536S), anti-pPKA (Thr197) (cat. 4781S), anti-Bcl-xL (cat. 2764S), anti- Bim (cat. 2933S), anti-cleaved caspase-3 (Asp175) (cat. 9664S), anti-SAPK/JNK (Thr183/Tyr185) (cat. 9251S), anti-p-p38 MAPK (Thr180/Tyr182) (cat. 4511S), anti-4E-BP1 (Thr37/46) (cat. 2855S), anti-PDK1 (Ser241) (cat. 3438S), anti-SDF1/CXCL12 (cat. 3740), anti-pNF-κB (Ser536) (cat.3033S), and anti-GSK3beta antibodies (Ser9) (cat. 5558S) were obtained from Cell Signaling Technology, Inc. (Danvers, MA).

Anti-pCREB (Ser133) (cat. ab32096), anti-TRAP (cat. ab191406), anti-Cathepsin K (cat. ab19027), anti-RANKL (ab9957), anti-M-CSF (ab52864), anti-MCP1 (ab73680), and anti-Osteocalcin antibodies (cat. ab93876) were obtained from Abcam (Cambridge, MA).

Anti-cMyc (cat. MA1-980) and anti-IL6 (AHC0762) antibodies were obtained from Thermo Fisher Scientific, Inc. (Waltham, MA).

### Compounds

Trametinib, selumetinib, PD184352, ulixertinib, SCH772984, GDC-0994, everolimus, and omipalisib were purchased from Selleck Chemicals (Houston, TX, USA). These chemicals were dissolved in DMSO.

### Quantitative PCR array

Total RNA was isolated using TRIzol (Thermo Fisher Scientific, Inc.) or RNeasy kit (Qiagen, Hilden, Germany) according to the manufacturer’s instructions. Single-stranded cDNA was synthesized from total RNA using the Maxima First Strand cDNA Synthesis Kit for RT-qPCR (Thermo Fisher Scientific, Inc.) according to the manufacturer’s instructions. Real-time RT-PCR was performed with QuantStudio 6 Flex (Applied Biosystems, Foster City, CA) using the Realplex system (Eppendorf, Hauppauge, NY) according to the manufacturer’s instructions. These data were analyzed in Microsoft Excel using the ΔΔCt method. All data were normalized to glyceraldehyde phosphate dehydrogenase gene expression values. The primers used for human genes and mouse genes are listed in Table S3. The results are expressed as the mean ± standard error (SE) from three independent experiments. *P* values were calculated using unpaired, two-tailed *t*-tests or one-way ANOVA with Dunnett’s multiple comparisons. Only significant values (*P* < 0.05) are depicted in the given heatmaps.

### RT² profiler PCR arrays

Eighty-four human inflammatory cytokine and cytokine receptor genes were profiled using a real-time RT² Profiler PCR Array (Catalog No. 330231; Qiagen, Valencia, CA) in pERK1/2-high MDA231 and pERK1/2-low MCF7 cell lines (Table S1). All samples passed quality control (PCR array reproducibility, PPC, GDC).

### Cell and tissue cultures

All cell lines (e.g., Hs578T, MDA-MB-157, MDA-MB-231, MCF7, MDA-MB-436, HCC1806, MC3T3-E1S4, and HEK293T) were obtained from the American Type Culture Center (ATCC, Manassas, VA) and cultured under conditions provided by the manufacturer. The MDA-MB231, MDA-MB-436, HEK293T, and MCF7 cell lines were cultured in DMEM supplemented with 10% FBS, 100 IU·mL^−1^ penicillin and 100 μg·mL^−1^ streptomycin (Thermo Fisher Scientific, Inc.). The Hs578T, MDA-MB-157, and HCC1806 cell lines were cultured in RPMI 1640 media (Thermo Fisher Scientific, Inc.) supplemented with 10% FBS and 100 IU·mL^–1^ penicillin/streptomycin. The MC3T3-E1S4 cell line was cultured in MEMalpha media (Thermo Fisher Scientific, Inc.) supplemented with 10% FBS and 100 IU·mL^–1^ penicillin/streptomycin.

### In vitro conditioned media-induced osteoclastogenesis

The TRAP assay was performed using an acid phosphatase staining kit (Sigma-Aldrich, St. Louis, MO) according to the manufacturer’s instructions. For osteoclastogenesis experiments, the indicated number of RAW264.7 mouse macrophage-like cells was seeded on a 12-well tissue culture plate with MEMalpha media supplemented with 10% FBS, 100 IU·mL^−1^ penicillin and 100 μg·mL^−1^ streptomycin. Raw cells were then treated with 20 ng·mL^−1^ mouse RANKL (R&D Systems, Inc., Minneapolis, MN) with or without conditioned media derived from MC3T3 cells. After 4 days of cell culture and osteoclast generation, the media were removed and washed three times with PBS. Cells were fixed with a fixing solution supplied by the manufacturer. The cells were incubated at 37 °C with a solution containing deionized water, Fast Garnet GBC, napthol phosphate, acetate, and tartrate for 1 h. The staining solution was removed, washed with PBS, and air-dried. TRAP-positive cells were collected using a Cytation 5 Imaging Reader (Biotek, Winooski, VT). TRAP-positive cells with three or more nuclei across the culture area were counted as multinucleated osteoclasts.

### Coculture experiments

MC3T3-E1 cells were seeded on six-well plates with osteogenic media comprised of MEMa media, 10% FBS, 1% penicillin/streptomycin and osteogenic factors (10 mmol·L^−1^ β-glycerol phosphate and 50 µg·mL^−1^ L-ascorbic acid). Then, 2.5 × 10^5^ cells were applied to each well. After 14 days, cancer cells (*n* = 200) were added to the plate and incubated for an additional 14 days in osteogenic media. The cells were fixed with 10% formaldehyde in PBS for 15 min at room temperature for further analysis.

### Alizarin red S staining

The fixed cells were stained with 1% (w/v) Alizarin Red S (Sigma-Aldrich, St. Louis, MO) solution for 20 min. The cells were then washed twice with PBS to elute the Alizarin Red S solution.

### Quantification of colony formation area

For measurement of tumor colony size, cells were stained with Hoechst 33342 for 5 min in PBS. Pictures of the stained cells were taken using a Cytation 5 Imaging Reader (Biotek, Winooski, VT). ImageJ analysis software (NIH, Bethesda, MD) was employed for analysis and colony quantification.

### MAPK phosphorylation array

Antibody arrays were performed to investigate activated kinases in osteoblastic MCF7 cells and osteolytic MDA231 cells. Two different MAPK phosphorylation assay kits were purchased from Raybiotech (Peachtree Corners, GA, USA) (cat no: AAH-MAPK-1–2) and Cell Signaling Technology, Inc. (Danvers, MA) (cat no: 7323). Antibody arrays were performed according to the manufacturer’s protocol.

### Western blot analysis

Western blotting was performed using established methods. Cells were washed with cold PBS and lysed in M-PER^™^ Mammalian Protein Extraction Reagent (Thermo Fisher Scientific, Inc.) with complete protease inhibitors (Roche Diagnostics, Indianapolis, IN) and PhosSTOP phosphatase inhibitors (Roche Diagnostics). Lysates were centrifuged at 13 000 r·min^−1^ for 10 min. Protein concentrations were determined via Bradford assay (Thermo Fisher Scientific, Inc.). Proteins were resolved by SDS-PAGE and transferred to a polyvinylidene difluoride membrane. The membrane was blocked with Membrane Blocking Solution (Thermo Fisher Scientific, Inc.) for 1 h at room temperature and incubated overnight with primary antibodies. Immunoblotting was performed for each antibody group per the manufacturer’s protocol.

### Histology

Harvested tissues were fixed for 1 day in 4% paraformaldehyde and PBS directly after sacrifice. After fixation, the bones were rinsed and decalcified with 10% EDTA (pH 7.2–7.4) for 2 weeks on a shaker. The specimens were subsequently processed to a thickness of 5 µm. Immunohistochemistry (IHC) was performed using the MACH 4 Universal HRP-Polymer system (Biocare Medical, LLC, Pacheco, CA). The slides were then incubated with the following primary antibodies of interest: pERK1/2 (1:500), pCaMKII (1:200), pAKT1 (1:200), pPKA (1:200), pCREB (1:200), pNF-kB (1:200), pcMyc (1:200), and RANKL (1:200). Reactions were then visualized with diaminobenzidine (DAB). The specimen sections were counterstained with hematoxylin. All staining was performed as instructed by the manufacturer. ImageJ software (NIH) was used to quantify the number of positively stained cells. In addition, multiplex immunohistochemistry was performed using fluorescein, Cyanine 3, and Cyanine 5 dyes (PerkinElmer, Boston, MA) according to the manufacturer’s instructions. Histologic images were captured using a Cytation 5 Imaging Reader (Biotek, Winooski, VT), and osteoblasts highly expressing pERK1/2 were manually counted by researchers (JB and LL) under blinded conditions.

### In vivo mRFP1-dnMERK1 MDA231 and mRFP-caMEK1 MCF7 cell line experiments

For evaluation of dnMEK1 and caMEK1 expression, plasmids were synthesized by PCR amplification with the primers listed in Table S4. The mRFP1-caMEK1 and mRFP-dnMEK1 plasmid constructs were designed using the NEBuilder^®^ HiFi DNA Assembly kit (NEB, Ipswich, MA). The plasmids were transformed into 50 μL of Stbl3-competent cells [10^7^ colony forming units (CFU) per μg] after heat shocking, and 10% of these bacteria was spread on LB plates inoculated with 100 μg·mL^−1^ ampicillin. Bacterial clones were counted to calculate ligation efficiency. The ratio of correctly assembled constructs was assessed by randomly sampling ten clones per reaction for plasmid purification and verification by sequencing. MDA231 cells (0.5 × 10^6^) carrying either the control mRFP1 (red fluorescent protein) or experimental dnMEK1-mRFP1 plasmid construct were intratibially injected into 10-week-old female nude mice. A total of 10^6^ MCF7 cells carrying control mRFP1 or experimental caMEK1-mRFP1 plasmids were injected into 10-week-old female nude mice via the intratibial approach. Animals were sacrificed at study endpoint. Upon sacrifice, body weight and tumor volume were recorded for each mouse, and the tibiae were collected and fixed in 10% neutral buffered formalin for 48 h and then decalcified in 10% EDTA for 2 weeks.

### Human bone implant experiments

Human bone was surgically isolated from the discarded femoral heads of patients who underwent TKA at the authors’ institution. The bones were subsequently cut into 5 mm × 5 mm × 5 mm sections. Mice who received these human bone implants were anesthetized by isoflurane prior to surgery. Using sterile surgical instruments, a 1 cm incision was created in the right and left dorsal flanks of 8-week-old nude mice. The bone cores were then coimplanted with human breast cancer cell lines (MCF7 and MDA-MB-231) or control media only. After these cells were implanted, the wound was closed with skin clips. Mice were subsequently provided analgesia per institutional guidelines and placed on a heating pad until fully awake. The bone implants were given 3 weeks to engraft in the mice.

### MEK inhibition experiments using trametinib

The effects of trametinib on cancer growth and osteolysis were investigated in ten MDA-MB-231-exposed mice. Breast cancer cell xenografts were implanted into the tibiae or implanted with live human bone into the right and left dorsal flanks of 9- to 10-week-old female Nu/J immunodeficient mice. For analysis of the effects of trametinib on the growth of breast cancer xenografts, mice bearing these xenografts were orally administered either vehicle (i.e., placebo) or 1 mg·kg^−1^ trametinib for 5 consecutive days each week over a 2-week period, which began 7 days (human bone model) or 14 days (tibia model) after initial tumor implantation. Animals were sacrificed 14 days (tibia model) or 21 days (human bone model) in the treatment period, at which time body weight and tumor volume were recorded and tumors were harvested for analysis.

### Microcomputed tomography (CT) analysis

Tibiae were stripped of soft tissue and stored in 10% formaldehyde in PBS for 48 hours and washed with PBS for microcomputed tomographic (CT) analyses. The bones were scanned using a Scanco μCT-35 machine (Scanco, Brutissellen, Switzerland). Volumetric regions within the endosteal borders of the distal femoral metaphysis, including the secondary spongiosa located 1 mm from the growth plate and extending 1 mm proximally, were selected and scanned at 12 μm resolution for trabecular analyses. Cortical morphometry was quantified and averaged volumetrically through 50 serial cross-sections 600 μm in size that extended distally from the diaphyseal midpoint between the proximal and distal growth plates. A customized thresholding technique was employed to generate optimal bone tissue segmentations. All micro-CT parameters were analyzed in both 2D and 3D according to the methods described by Bouxsein.^58^

### Statistical analysis

All statistical analyses were performed using GraphPad Prism software versions 7 and 8 (GraphPad Software, San Diego, CA). All results are expressed as the mean ± SE using triplicate measurements across experiments. Differences between experimental groups were analyzed by paired Student’s *t* tests or one-way analysis of variance (ANOVA) followed by Dunnett’s post-test. Differences were reported as statistically significant for *P* values < 0.05. Statistical significance was reported as follows: * denotes *P* < 0.05; ** denotes *P* < 0.01; *** denotes *P* < 0.001; and **** denotes *P* < 0.000 1.

### Study approval

All murine experiments were approved by the Yale University School of Medicine Institutional Animal Care and Use Committee (IACUC; Number: 2020-20129). All human bone tissue utilized in these experiments was obtained in compliance with NIH regulations and institutional guidelines and approved by the Institutional Review Board of the Yale University School of Medicine (2000021232) and the Columbia University Medical Center (AAAD0975(Y5M00)).

## Supplementary information

Legends for supplementary figures and tables

Supplementary figures 1-6 and Tables 1-4

Suuplementary table 5

## Data Availability

The cell lines described in this article can be obtained through a material transfer agreement with the authors and Yale University.
