# Supplementary material for: Inflammatory conversion of quiescent osteoblasts by metastatic breast cancer cells through pERK1/2 aggravates cancer-induced bone destruction
Source: Bone Res. 2021 Sep 29;9:43. doi: 10.1038/s41413-021-00158-w (PMC8481290; doi:10.1038/s41413-021-00158-w)
Supplement: Supplementary file 1 — Legends for supplementary figures and tables [file 41413_2021_158_MOESM1_ESM.docx]

Supplementary Materials:

**Figure S1.** The ERK pathway is activated in aggressive osteolytic cancer cells. (**a**) Densitometric analysis of the MAPK array. For each protein, data from MDA231 samples are documented as fold changes in expression compared to the corresponding values from MCF7 samples. (**b**) Densitometric analysis of the MAPK array. For each protein, data from MDA231 samples are documented as fold changes in expression compared to corresponding values from MCF7 samples.

**Figure S2.** Eighty-four genes encoding human inflammatory cytokines and receptors were profiled using the real-time RT² Profiler PCR Array. The fold change was calculated using the formula 2−^△△CT^. (**a**) Fold change in expression of genes in pERK1/2-high MDA231 cells per pERK1/2-low MCF7 cells. (**b**) Fold change in expression of genes in DMSO-treated MDA231 cells per MEK inhibitor-treated MDA231 cells.

**Figure S3.** Increase in osteoclastogenic inflammatory cytokine expression by MEK1/ERK1/2 activation in BCCs and Obs. (**a**) Immunoblot analyses of RFP, RFP-tagged dnMEK1, RFP-tagged caMEK1 and pERK1/2 expression in Obs. (**b**) Immunoblot analyses of ERK1/2 expression in RFP-tagged dnMEK1 and levels of pERK1/2 in MCF7 cells. ERK1/2 activation in MDA231 cells was reduced in the presence of the dnMEK1 construct. (**c**) Differential heat map of upregulated expression of inflammatory osteoclastogenic cytokines and chemokines in the RFP-MCF7 and caMEK1-MCF7 cells. (**d**) Expression of the osteoclastogenic inflammatory cytokines IL-1β and IL-8 was increased following ERK1/2 activation in MCF7 cells. *P* values were calculated by two-tailed Student’s *t*-test. (**e**) Immunoblot analyses of RFP, RFP-tagged dnMEK1, RFP-tagged caMEK1 and pERK1/2 expression in MC3T3 cells. (**f**) Heat map illustrative of elevated osteoclastogenic inflammatory cytokine expression in the pERK1/2-high MC3T3 cells. (**g**) ERK1/2 activation reduced the mineralization of MC3T3 cells by inhibiting osteogenic gene expression.

**Figure S4.** pERK1/2+ Obs secrete regulatory molecules to promote BCC growth. (**a**) Increased BCC growth was observed in the presence of osteoblasts highly expressing ERK1/2. Scale bar: 200 μm. (**b**) Mean colony area size was computed from images from the experiment in panel (**a**). *P* values were calculated using a two-tailed Student’s *t*-test. (**c**) Differential heat map of upregulated inflammatory cytokine, chemokine, and growth factor expression in the pERK1/2-high osteoblasts. (**d**) Differential heat map of downregulated inflammatory cytokine, chemokine, and growth factor expression in the dnMEK1-expressing osteoblasts cocultured with pERK1/2-high BCCs.

**Figure S5. Trametinib inhibits BCC-induced expression of proinflammatory molecules in the bone microenvironment.** (**a**) Heat map illustrating decreased expression of inflammatory cytokines in BCCs after in vivo trametinib treatment. (**b**) Heat map illustrating the downregulated expression of inflammatory cytokines, chemokines, and growth factors in osteoblasts cocultured with clinically aggressive MDA231 cells after in vivo trametinib treatment.

**Figure S6. pERK1/2-high Obs-derived CM induces preosteoclast cell proliferation but reduces osteoclast formation in vitro.** (**a**) Schematic representation of the experiment. (**b**) Quantification of osteoclast formation by the effect of Obs-derived CM. MC3T3-derived CM was treated with preosteoclast cells. (**c**) Quantification of osteoclast formation by the effect of BCC/Ob-derived CM. MDA231-derived CM was treated with MC3T3 cells, and then, MC3Ts-derived CM was treated with preosteoclast cells. (**d**) Trametinib inhibits osteoclast formation in low populations of preosteoclast cells. Decreased TRAP+ multinuclear cells were observed in the presence of trametinib. Scale bar: 1 mm. TRAP+ multinuclear cell number calculated from images from the experiment in each panel. *P* values were calculated using the two-tailed Student’s *t*-test.

**Tables**

**Table S1. Breast cancer cell lines (ATCC).**

**Table S2. Eighty-four genes encoding human inflammatory cytokines and receptors were profiled using the real-time RT² Profiler PCR Array. The fold change was calculated using the formula 2^−△△CT^.**

**Table S3. Activation of kinases*, transcription factors†, and inflammatory cytokine expression‡ in osteolytic breast cancer cells.**

**Table S4. The species-specific primers for gene expression analyses of cytokines, chemokines, and growth factors.**

**Table S5. Primers used to construct the dnMEK1 and caMEK1 plasmids.**
