# Supplementary material for: Inflammatory conversion of quiescent osteoblasts by metastatic breast cancer cells through pERK1/2 aggravates cancer-induced bone destruction
Source: Bone Res. 2021 Sep 29;9:43. doi: 10.1038/s41413-021-00158-w (PMC8481290; doi:10.1038/s41413-021-00158-w)
Supplement: Supplementary file 2 — Supplementary figures 1-6 and Tables 1-4 [file 41413_2021_158_MOESM2_ESM.pdf]

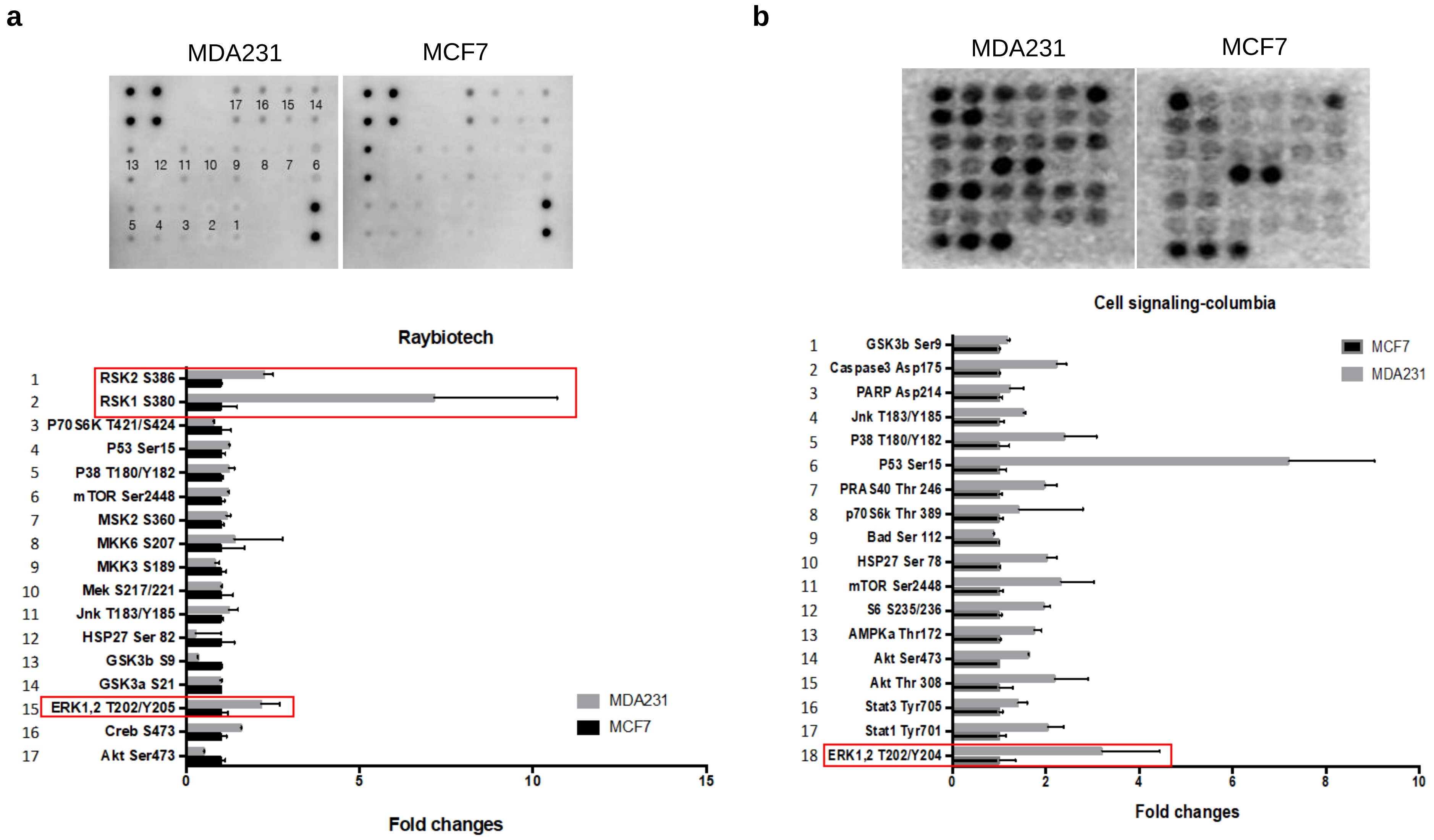
a
MDA231
MCF7
17 16 15 14
13 12 11 10 9 8 7 6
5 4 3 2 1
Raybiotech
1 RSK2 S386
2 RSK1 S380
3 P70S6K T421/S424
4 P53 Ser15
5 P38 T180/Y182
6 mTOR Ser2448
7 MSK2 S360
8 MKK6 S207
9 MKK3 S189
10 Mek S217/221
11 Jnk T183/Y185
12 HSP27 Ser 82
13 GSK3b S9
14 GSK3a S21
15 ERK1,2 T202/Y205
16 Creb S473
17 Akt Ser473
MDA231
MCF7
0
5
10
15
Fold changes
b
MDA231
MCF7
Cell signaling-columbia
1 GSK3b Ser9
2 Caspase3 Asp175
3 PARP Asp214
4 Jnk T183/Y185
5 P38 T180/Y182
6 P53 Ser15
7 PRAS40 Thr 246
8 p70S6k Thr 389
9 Bad Ser 112
10 HSP27 Ser 78
11 mTOR Ser2448
12 S6 S235/236
13 AMPKa Thr172
14 Akt Ser473
15 Akt Thr 308
16 Stat3 Tyr705
17 Stat1 Tyr701
18 ERK1,2 T202/Y204
MCF7
MDA231
0
2
4
6
8
10
Fold changes

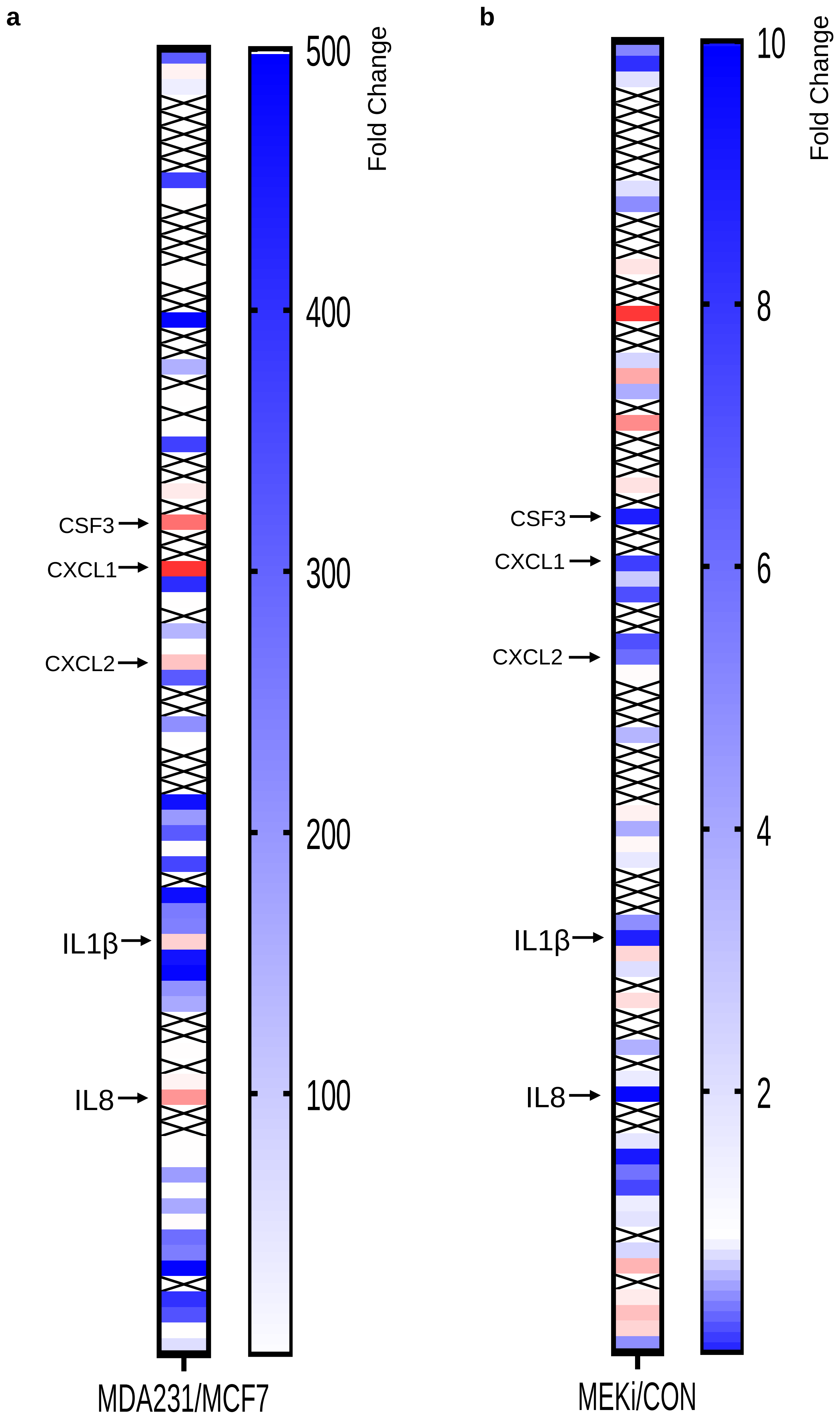
a
CSF3
CXCL1
CXCL2
IL1β
IL8
500
400
300
200
100
Fold Change
MDA231/MCF7
b
CSF3
CXCL1
CXCL2
IL1β
IL8
10
8
6
4
2
Fold Change
MEKi/CON

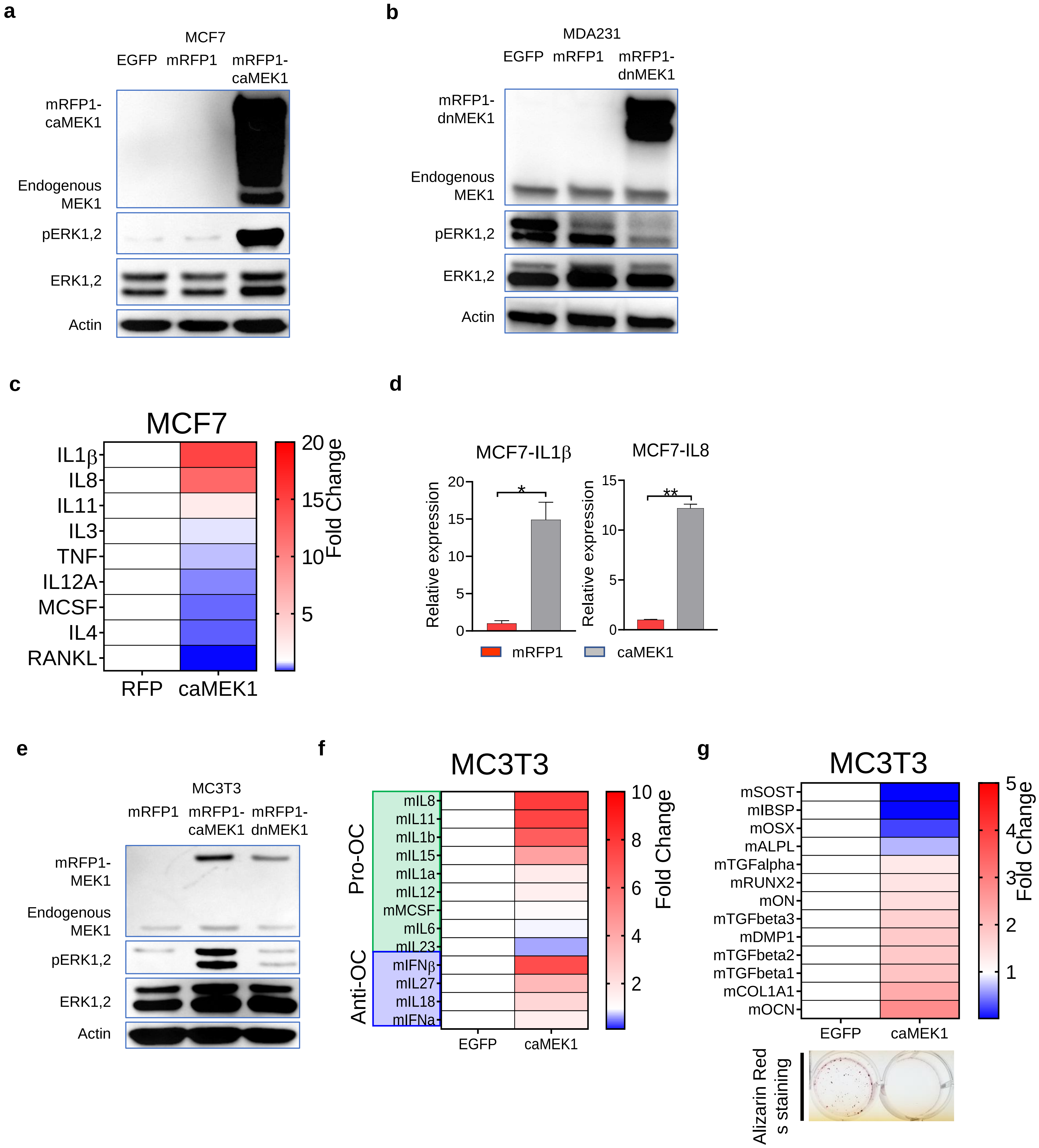
a
MCF7
EGFP
mRFP1
mRFP1-caMEK1
mRFP1-caMEK1
Endogenous MEK1
pERK1,2
ERK1,2
Actin
b
MDA231
EGFP
mRFP1
mRFP1-dnMEK1
mRFP1-dnMEK1
Endogenous MEK1
pERK1,2
ERK1,2
Actin
c
MCF7
IL1β
IL8
IL11
IL3
TNF
IL12A
MCSF
IL4
RANKL
RFP
caMEK1
Fold Change
20
15
10
5
d
MCF7-IL1β
MCF7-IL8
Relative expression
*
**
mRFP1
caMEK1
e
MC3T3
mRFP1
mRFP1-caMEK1
mRFP1-dnMEK1
mRFP1-MEK1
Endogenous MEK1
pERK1,2
ERK1,2
Actin
f
MC3T3
Pro-OC
Anti-OC
mIL8
mIL11
mIL1b
mIL15
mIL1a
mIL12
mMCSF
mIL6
mIL23
mIFNβ
mIL27
mIL18
mIFNa
EGFP
caMEK1
Fold Change
g
MC3T3
mSOST
mIBSP
mOSX
mALPL
mTGFalpha
mRUNX2
mON
mTGFbeta3
mDMP1
mTGFbeta2
mTGFbeta1
mCOL1A1
mOCN
EGFP
caMEK1
Fold Change
Alizarin Red s staining

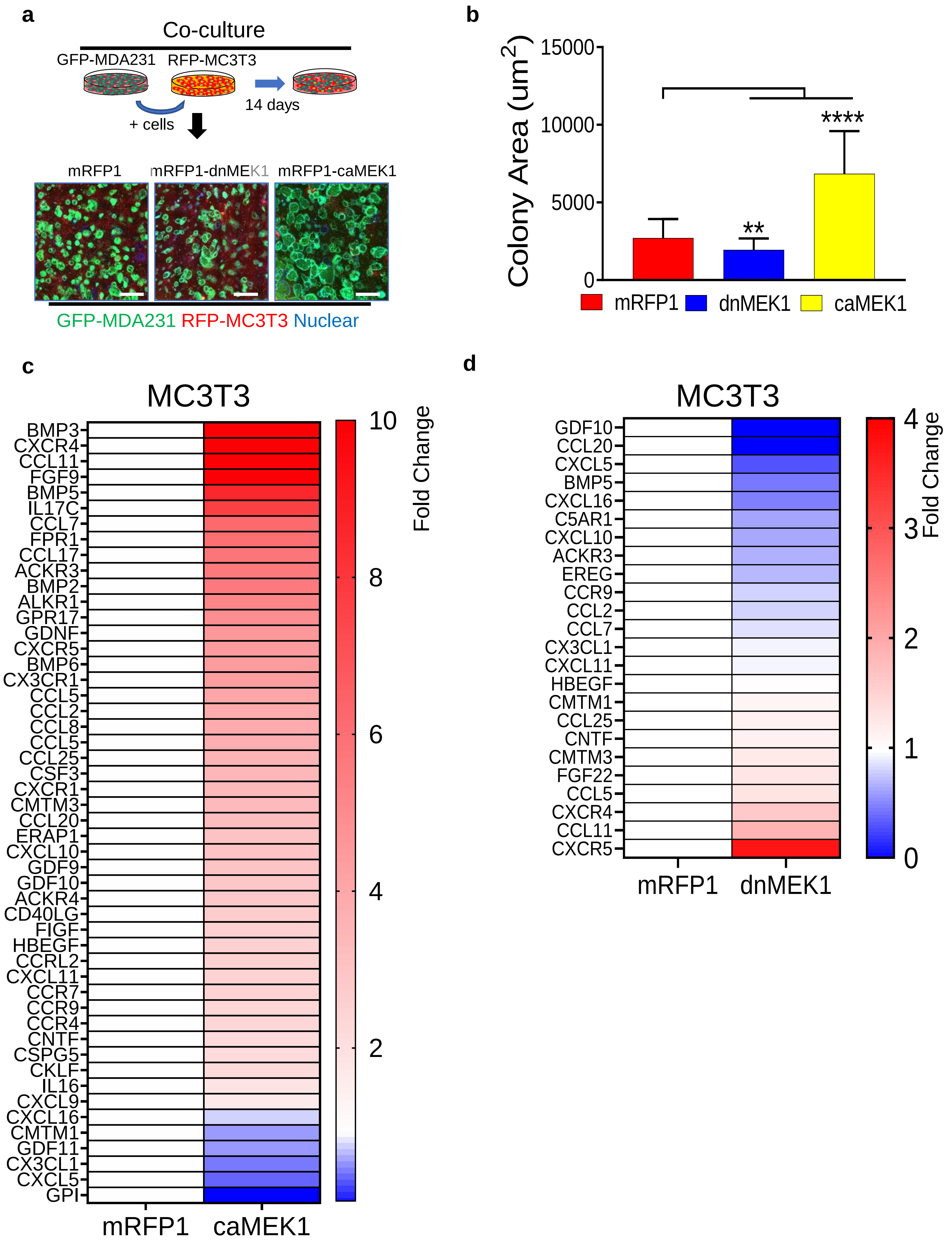
a
Co-culture
GFP-MDA231
RFP-MC3T3
14 days
+ cells
mRFP1
mRFP1-dnMEK1
mRFP1-caMEK1
GFP-MDA231 RFP-MC3T3 Nuclear
b
Colony Area (um2)
15000
10000
5000
0
**
****
mRFP1
dnMEK1
caMEK1
c
MC3T3
BMP3
CXCR4
CCL11
FGF9
BMP5
IL17C
CCL7
FPR1
CCL17
ACKR3
BMP2
ALKR1
GPR17
GDNF
CXCR5
BMP6
CX3CR1
CCL5
CCL2
CCL8
CCL5
CCL25
CSF3
CXCR1
CMTM3
CCL20
ERAP1
CXCL10
GDF9
GDF10
ACKR4
CD40LG
FIGF
HBEGF
CCRL2
CXCL11
CCR7
CCR9
CCR4
CNTF
CSPG5
CKLF
IL16
CXCL9
CXCL16
CMTM1
GDF11
CX3CL1
CXCL5
GPI
mRFP1
caMEK1
10
8
6
4
2
Fold Change
d
MC3T3
GDF10
CCL20
CXCL5
BMP5
CXCL16
C5AR1
CXCL10
ACKR3
EREG
CCR9
CCL2
CCL7
CX3CL1
CXCL11
HBEGF
CMTM1
CCL25
CNTF
CMTM3
FGF22
CCL5
CXCR4
CCL11
CXCR5
mRFP1
dnMEK1
4
3
2
1
0
Fold Change

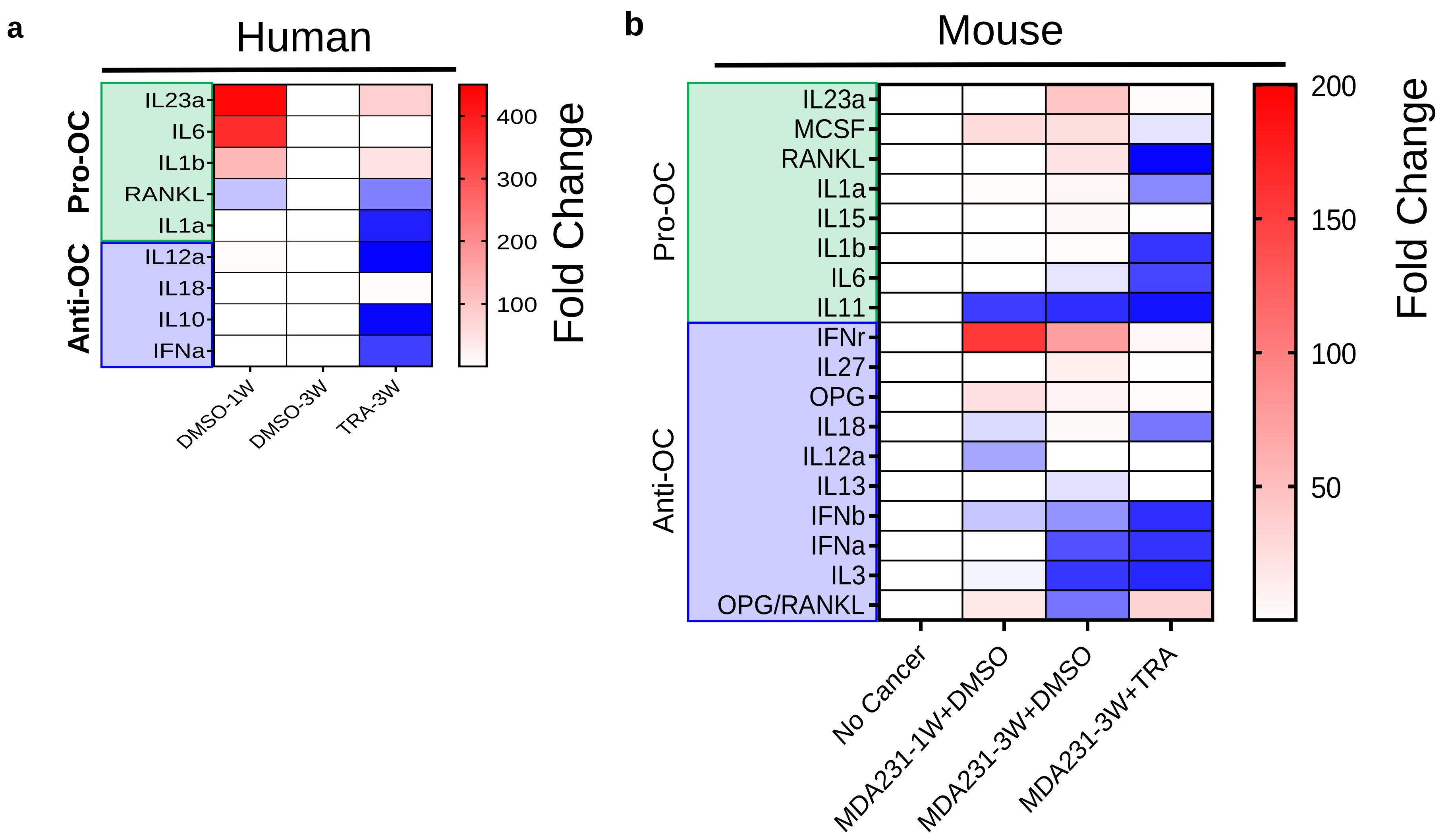
a
Human
Pro-OC
Anti-OC
IL23a
IL6
IL1b
RANKL
IL1a
IL12a
IL18
IL10
IFNa
DMSO-1W
DMSO-3W
TRA-3W
400
300
200
100
Fold Change
b
Mouse
Pro-OC
Anti-OC
IL23a
MCSF
RANKL
IL1a
IL15
IL1b
IL6
IL11
IFNr
IL27
OPG
IL18
IL12a
IL13
IFNb
IFNa
IL3
OPG/RANKL
No Cancer
MDA231-1W+DMSO
MDA231-3W+DMSO
MDA231-3W+TRA
200
150
100
50
Fold Change

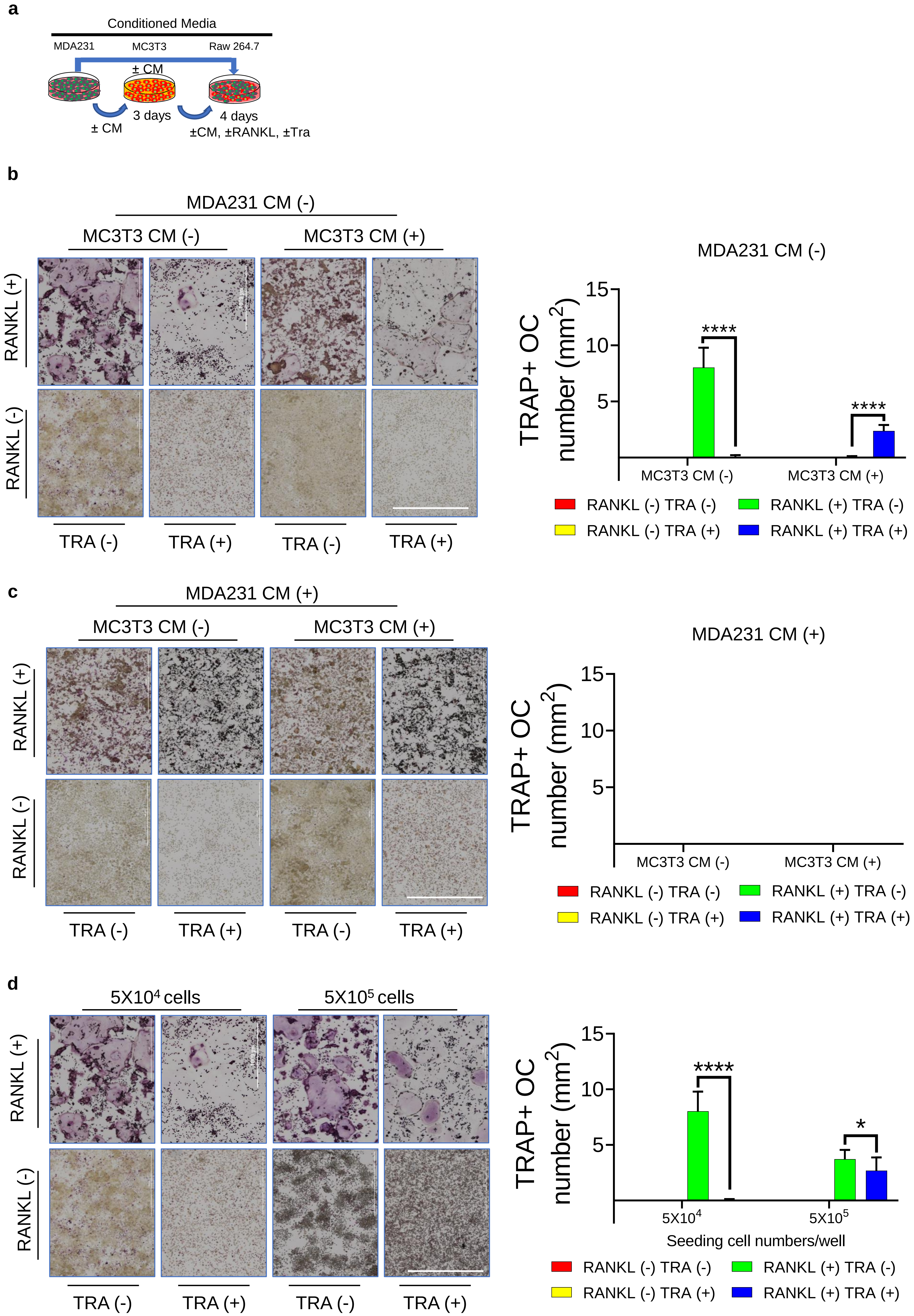
a
Conditioned Media
MDA231
MC3T3
Raw 264.7
± CM
3 days
± CM
4 days
±CM, ±RANKL, ±Tra
b
MDA231 CM (-)
MC3T3 CM (-)
MC3T3 CM (+)
RANKL (+)
RANKL (-)
TRA (-)
TRA (+)
TRA (-)
TRA (+)
MDA231 CM (-)
TRAP+ OC number (mm2)
15
10
5
****
****
MC3T3 CM (-)
MC3T3 CM (+)
RANKL (-) TRA (-)
RANKL (+) TRA (-)
RANKL (-) TRA (+)
RANKL (+) TRA (+)
c
MDA231 CM (+)
MC3T3 CM (-)
MC3T3 CM (+)
RANKL (+)
RANKL (-)
TRA (-)
TRA (+)
TRA (-)
TRA (+)
MDA231 CM (+)
TRAP+ OC number (mm2)
15
10
5
MC3T3 CM (-)
MC3T3 CM (+)
RANKL (-) TRA (-)
RANKL (+) TRA (-)
RANKL (-) TRA (+)
RANKL (+) TRA (+)
d
5X10^4 cells
5X10^5 cells
RANKL (+)
RANKL (-)
TRA (-)
TRA (+)
TRA (-)
TRA (+)
TRAP+ OC number (mm2)
15
10
5
****
*
5X10^4
5X10^5
Seeding cell numbers/well
RANKL (-) TRA (-)
RANKL (+) TRA (-)
RANKL (-) TRA (+)
RANKL (+) TRA (+)

Breast cancer cell lines (ATCC)

Name	ER	Histology	Tumor Source	Mutant Gene	Morphology
MCF7	ER positive	Adenocarcinoma	metastasis, pleural effusion	CDKN2A (c.1_471del471), PIK3CA (p.E545K)	Epithelial
HCC1806	TRIPLE NEGATIVE	Primary acantholytic squamous cell carcinoma	primary	CDKN2A (c.1_471del471), KDM6A (c.444_564del121), STK11 (c.1109_1302del194), TP53 (p.T256fs*90)	Basal-Like Morphology
MDA-MB-231	TRIPLE NEGATIVE	Adenocarcinoma	metastasis, pleural effusion	BRAF (p.G464V), CDKN2A(c.1_471del471), KRAS (p.G13D), NF2 (p.E231*), TP53 (p.R280K)	Mesenchymal-Like Morphology
MDA-MB-436	TRIPLE NEGATIVE	Adenocarcinoma	metastasis, pleural effusion	BRCA1(c.5277+1G>A), RB1 (p.G203fs*9)	Mesenchymal-Like Morphology
MDA-MB-157	TRIPLE NEGATIVE	Medulallary carcinoma	metastasis, pleural effusion	NF1(p.S2751fs*27), TP53 (p.A88fs*52)	Mesenchymal-Like Morphology
Hs 578T	TRIPLE NEGATIVE	Carcinoma	primary	CDKN2(c.1_471del471), HRAS (p.G12D) , PIK3R1(p.N453_T454insN), TP53 (p.V157F)	Mesenchymal-Like Morphology

Well	Gene Symbol	Fold Difference		Well	Gene Symbol	Fold Difference	
		MDA231/MCF7	MDA231-MEKi/Con			MDA231/MCF7	MDA231-MEKi/Con
A01	AIMP1	0.36	0.52	D07	CXCL9	N.D	N.D
A02	BMP2	25.07	0.19	D08	CXCR1	0.56	N.D
A03	C5	0.93	0.88	D09	CXCR2	1.40	0.71
A04	CCL1	N.D	N.D	D10	FASLG	N.D	N.D
A05	CCL11	N.D	N.D	D11	IFNA2	N.D	N.D
A06	CCL13	N.D	N.D	D12	IFNG	N.D	N.D
A07	CCL15	N.D	N.D	E01	IL10RA	0.06	N.D
A08	CCL16	N.D	N.D	E02	IL10RB	0.60	1.46
A09	CCL17	0.24	N.D	E03	IL13	0.35	0.67
A10	CCL2	1.02	0.87	E04	IL15	3.12	1.28
A11	CCL20	N.D	0.55	E05	IL16	0.27	0.91
A12	CCL22	N.D	N.D	E06	IL17A	N.D	N.D
B01	CCL23	N.D	N.D	E07	IL17C	0.05	N.D
B02	CCL24	N.D	N.D	E08	IL17F	0.48	N.D
B03	CCL26	1.04	1.95	E09	IL1A	0.50	0.56
B04	CCL3	N.D	N.D	E10	IL1B	89.20	0.13
B05	CCL4	N.D	N.D	E11	IL1R1	0.07	2.43
B06	CCL5	0.02	8.05	E12	IL1RN	0.02	0.87
B07	CCL7	N.D	N.D	F01	IL21	0.57	N.D
B08	CCL8	N.D	N.D	F02	IL27	0.66	2.21
B09	CCR1	0.69	0.83	F03	IL3	N.D	N.D
B10	CCR2	N.D	4.01	F04	IL33	N.D	N.D
B11	CCR3	2.11	0.68	F05	IL5	3.25	0.69
B12	CCR4	N.D	N.D	F06	IL5RA	N.D	N.D
C01	CCR5	1.33	5.07	F07	IL7	24.39	0.92
C02	CCR6	0.25	N.D	F08	IL8	207.36	0.03
C03	CCR8	N.D	N.D	F09	IL9	N.D	N.D
C04	CD40LG	N.D	N.D	F10	IL9R	N.D	N.D
C05	CSF1	41.21	2.00	F11	LTA	1.31	0.90
C06	CSF2	N.D	N.D	F12	LTB	2.54	0.10
C07	CSF3	278.98	0.12	G01	MIF	0.61	0.45
C08	CX3CL1	N.D	N.D	G02	NAMPT	1.78	0.28
C09	CX3CR1	N.D	N.D	G03	OSM	0.66	0.93
C10	CXCL1	398.38	0.25	G04	SPP1	5.88	0.89
C11	CXCL10	0.17	0.79	G05	TNF	0.43	N.D
C12	CXCL11	2.88	0.31	G06	OPG	0.49	0.84
D01	CXCL12	N.D	N.D	G07	TNFSF10	0.01	3.62
D02	CXCL13	0.71	N.D	G08	RANKL	N.D	N.D
D03	CXCL2	654.39	0.32	G09	TNFSF13	0.19	1.68
D04	CXCL3	116.89	0.43	G10	TNFSF13B	0.32	3.25
D05	CXCL5	0.35	1.13	G11	TNFSF4	1.03	2.51
D06	CXCL6	N.D	N.D	G12	VEGFA	0.87	0.56

Sample ID	Phenotype	Location	pERK1/2[*]	p-CaMKII[*]	pAKT1[†]	P-PKA[†]	pCREB[†]	P-NF-kB[†]	P-cMyc[†]	RANKL[‡]
BCBM011316b	Osteolytic	Femur	++/30%	+++/50%	-	+/10%	++/30%	+/40%	+++/90%	++/90%
BCBM012815b	Mixed	Femur	++/60%	+++/100%	-	++/100%	++/80%	-	+++/90%	++/100%
BCBM012815S	Mixed	Femur	++/25%	+/80%	-	++/40%	+/10%	++/40%	++/60%	++/90%
BCBM020516	Mixed	Femur	+++/70%	+/90%	+-/50%	+/70%	++/60%	+/30%	+/20%	+++/100%
BCBM022015B1	Osteolytic	Femur	++/10%	++/100%	-	++/100%	+/25%	+/60%	+-/70%	+++/100%
BCBM41316-A	Osteoblastic	Femur	++/70%	++/10%	-	+++/80%	++/50%	-	++/80%	+++/100%
BCBM072215T+	Mixed	Femur	++/60%	+/100%	-	++/100%	+-/40%	-	++/100%	+/80%
BCBM111314-2	Mixed	Femur	-	++/20%	-	++/80%	+++/40%			++/90%
Breastmet1	Osteolytic	Femur	++80%	++/70%	-	++/70%	++/90%	+++/50%	++/50%	++/90%
Breastmet2	Osteolytic	Femur	+++/60%	+++/60%	-	++/60%	+++/10%	+++/70%	++/50%	++/80%
157283	Osteoblastic	Femur	-	ND	ND	ND	-	ND	ND	ND
157288	Mixed	Femur	+++/50%	ND	ND	ND	++/40%	ND	ND	ND
157291	Mixed	Femur	+++/80%	ND	ND	ND	+++/70%	ND	ND	ND
157293	Osteoblastic	Femur	+/10%	ND	ND	ND	-	ND	ND	ND
157289	Osteolytic	Femur	+/10%	ND	ND	ND	+++/10%	ND	ND	ND
157297	Mixed	Femur	++/80%	ND	ND	ND	+/30%	ND	ND	ND
157300	Osteoblastic	Femur	+++/100%	ND	ND	ND	ND	ND	ND	ND
157284	Mixed	Femur	++/30%	ND	ND	ND	-	ND	ND	ND
157286	Osteolytic	Femur	++/80%	ND	ND	ND	+++/50%	ND	ND	ND

Human		Mouse	
Primer Name	Sequence (5' to 3')	Primer Name	Sequence (5' to 3')
hACKR1-F624	GCAGGCCACACACACTGTAG	mACKR1-F678	CAAGGGGCTGAAGATAGCAC
hACKR1-R824	AACAGCAACAGCTTGGACCT	mACKR1-R867	GGCTTCTGTCACATTCAGCA
hACKR2-F344	TCTTGTGCAAGATGGTGAGC	mACKR2-F177	CTCCGTAGTTGTGGCGATTT
hACKR2-R541	CCATATCAGGGATGGAGACG	mACKR2-F630	CTTCCAGCTGAACCTTCTGG
hACKR3-F277	GTTGTCCTCACCATCCCAGT	mACKR2-R324	GCCTAGAATCCATCCGTGAA
hACKR3-R416	CTCATGCACGTGAGGAAGAA	mACKR2-R823	GCAACGAGTGCAGAAACAAG
hACKR4-F153	TTTCGTCATTGGACTTGCAG	mACKR3-F633	CATGGAGCTGGTCTCTGTCA
hACKR4-R302	GCATTAACAGCCCAAAAAGG	mACKR3-R796	GCCAACATACCAGGAAGACC
hADIPOQ-F47	ATGACCAGGAAACCACGACT	mADIPOQ-F9	GTTGCAAGCTCTCCTGTTCC
hADIPOQ-R244	CACCGATGTCTCCCTTAGGA	mADIPOQ-R202	TCTCTCCAGGAGTGCCATCT
hAMH-F576	CACCCGCTACCTGGTGTTAG	mAMH-F544	CAGAACCTCTGCCCTACTCG
hAMH-R738	GGTCATCCGTGTGAAGCAG	mAMH-R710	CGGGAATCAGAGCCAAATAG
hBDNF-F164	GCTTGACATCATTGGCTGAC	mBDNF-F79	GCGCCCATGAAAGAAGTAAA
hBDNF-R320	AGAAGAGGAGGCTCCAAAGG	mBDNF-R234	TTCGATGACGTGCTCAAAAG
hBMP10-F546	CATGCTGGTCTTGGTGTCTG	mBMP10-F1047	CCGGGGTGTGTGTAACTACC
hBMP10-R747	ATGCTTATTCTGGGCACTGG	mBMP10-R1248	AGACACAGCCATCCCTTCAT
hBMP1-F702	TTCCATCGTTCGTGAGAACA	mBMP1-F828	CTATGCCCGGAACACATTCT
hBMP1-R885	CACCCCGTTCACCTCATACT	mBMP1-R1004	GTGCTGTCTTGGAGGGTCTC
hBMP2-F413	CTATCCCCACGGAGGAGTTT	mBMP2-F619	GCTCCACAAACGAGAAAAGC
hBMP2-R590	ACCAACCTGGTGTCCAAAAG	mBMP2-R996	AGCAAGGGGAAAAGGACACT
hBMP3-F1137	TGCAGATATTGGCTGGAGTG	mBMP3-F500	TCCTCAAATCCAACCAAAGC
hBMP3-R1317	TGGTACACAGCAAGGCTCAG	mBMP3-R682	TCCTCTTGGGTAGCTCGTGT
hBMP4-F296	TCCACAGCACTGGTCTTGAG	mBMP4-F716	TGACTCACCTCCACCAGACA
hBMP4-R443	GGGATGCTGCTGAGGTTAAA	mBMP4-R888	ATGCTTGGGACTACGTTTGG
hBMP5-F255	CAATGCCATGACCAATGAAG	mBMP5-F1193	ACCATGCCATAGTCCAGACC
hBMP5-R425	AGAGGAGGACTCTGGGTGGT	mBMP5-R1359	GCAGCCACACGAACGTACTA
hBMP6-F1300	AAGGGCTATGCTGCCAATTA	mBMP6-F534	CTCCTTGAACCGCAAGAGTC
hBMP6-R1465	GAACCGAGATGGCATTTAGC	mBMP6-R683	TGTGGGGAGAACTCCTTGTC
hBMP7-F416	TCGTGGAACATGACAAGGAA	mBMP7-F726	GGGCTTACAGCTCTCTGTGG
hBMP7-R578	CTGATCCGGAACGTCTCATT	mBMP7-R873	CCGGATACTACGGAGATGGA
hBMP8B-F853	CTCCCAGGGATCTTTGATGA	mBMP8B-F1037	CTATGCAGGCCCTGGTACAT
hBMP8B-R1003	AGCACTCCCCCTCACAGTAA	mBMP8B-R1186	AGGCCTGGACTACCATGTTG
hC5AR1-F840	GCTGGACTCCCTGTGTGTCT	mC5AR1-F499	CTCACCATTCCATCCTTCGT
hC5AR1-R991	CCCTAACCACGGACTCTTCA	mC5AR1-R653	GTGAGCAGAGGCAACACAAA
hC5-F1087	CTGAAGCCTGGGATTCCATA	mC5-F681	TGTCTTGCCACGATTCTCTG
hC5-R1287	CGTCACTCCAGATGGGAGAT	mC5-R877	GTGTGGCTTTGTGCATCATC
hCCL1-F72	GAGCATGCAGGTACCCTTCT	mCCL1-F74	GCATGCTTACGGTCTCCAAT
hCCL1-R217	AGGCCTCTTTGCCTCTCTTC	mCCL1-R232	TAGTTGAGGCGCAGCTTTCT
hCCL2-F89	TCAGCCAGATGCAATCAATG	mCCL2-F129	CCCAATGAGTAGGCTGGAGA
hCCL2-R223	AGATCTCCTTGGCCACAATG	mCCL2-R284	ATTTGGTTCCGATCCAGGTT
hCCL3-F53	GCAACCAGTTCTCTGCATCA	mCCL3-F98	CCTGCTGCTTCTCCTACAGC
hCCL3-R207	GCTTCGCTTGGTTAGGAAGA	mCCL3-R247	CTTGGACCCAGGTCTCTTTG
hCCL4-F10	TGCGTGACTGTCCTGTCTCT	mCCL4-F89	CTCCCACTTCCTGCTGTTTC
hCCL4-R146	ACCACAAAGTTGCGAGGAAG	mCCL4-R237	CTCACTGGGGTTAGCACAGA
hCCL5-F24	CGCTGTCATCCTCATTGCTA	mCCL5-F20	CCCTCACCATCATCCTCACT
hCCL5-R173	GAGCACTTGCCACTGGTGTA	mCCL5-R171	GCACTTGCTGCTGGTGTAGA
hCCL7-F1	ATGAAAGCCTCTGCAGCACT	mCCL7-F85	AATGCATCCACATGCTGCTA
hCCL7-R150	GCTCTCCAGCCTCTGCTTAG	mCCL7-R254	ATAGCCTCCTCGACCCACTT
hCCL8-F84	TTCCATTCCAATCACCTGCT	mCCL8-F50	TCAGCCCAGAGAAGCTGACT
hCCL8-R232	GGTCAGCACAGACCTCCTTG	mCCL8-R196	TCTGGAAAACCACAGCTTCC
hCCL11-F75	AGCTTCTGTCCCAACCACCT	mCCL11-F36	CACGGTCACTTCCTTCACCT
hCCL11-R214	TATCCTTGGCCAGTTTGGTC	mCCL11-R187	CTATGGCTTTCAGGGTGCAT
hCCL13-F86	CTTACCACCCCTCAGAGTGC		
hCCL13-F87	CGTCCCATCTACTTGCTGCT		
hCCL13-R233	GGGTTGGTACAGACGGAATG		
hCCL13-R240	CTTCTCCTTTGGGTCAGCAC		
hCCL15-F141	TCACTTTGCTGCTGACTGCT		
hCCL15-R291	ACTGGGTTTGGCACAGACTT		
hCCL16-F162	CAGAAAGGCCCTCAACTGTC		
hCCL16-R307	TGGACAAGTTCCTGGTAGGC		
hCCL17-F47	CTTCTCTGCAGCACATCCAC	mCCL17-F65	TGCTTCTGGGGACTTTTCTG
hCCL17-R209	CTGCCCTGCACAGTTACAAA	mCCL17-R211	CATCCCTGGAACACTCCACT
hCCL18-F83	AGCTCTGCTGCCTCGTCTAT		
hCCL18-R232	CCCACTTCTTATTGGGGTCA		
hCCL19-F95	CTGTGACCCAGAAACCCATC	mCCL19-F141	GAAAGCCTTCCGCTACCTTC
hCCL19-R252	CTGGATGATGCGTTCTACCC	mCCL19-R304	TGCTGTTGCCTTTGTTCTTG
hCCL20-F79	GCAAGCAACTTTGACTGCTG	mCCL20-F249	CACCCAGTTCTGCTTTGGAT
hCCL20-R228	ATTTGCGCACACAGACAACT	mCCL20-F93	CGACTGTTGCCTCTCGTACA
hCCL21-F30	TATCCTGGTTCTGGCCTTTG	mCCL21-F222	ATGTGCAAACCCTGAGGAAG
hCCL21-R170	CAGCCTAAGCTTGGTTCCTG	mCCL21-F399	TCCTCTTGAGGGCTGTGTCT
hCCL22-F16	ACTGCACTCCTGGTTGTCCT	mCCL22-F40	CTTCTTGCTGTGGCAATTCA
hCCL22-R180	GCAGGAGTCTGAGGTCCAGT	mCCL22-R219	TCGGTTCTTGACGGTTATCA
hCCL23-F202	AGTGCTGACTGCTGCATCTC		
hCCL23-R382	GCTTCAGCATTCTCATGCAA		
hCCL24-F75	CTCTGTGGTCATCCCCTCTC	mCCL24-F77	CTGTGACCATCCCCTCATCT
hCCL24-R254	TACCTCTGGACCCACTCCTG	mCCL24-R258	TATGTGCCTCTGAACCCACA
hCCL25-F212	ACAGGAAGGTGTGTGGGAAC	mCCL25-F173	GCAACCTACGTGCTGTGAGA
hCCL25-R409	TACTGCTGCTGATGGGATTG	mCCL25-R359	GGGTTCTCCACCTTGGACTT
hCCL26-F42	CCTCCTGAGTCTCCACCTTG	mCCL26-F23	TTGTTCTCCTGGCCATCTTC
hCCL26-R176	TGGGAGCAGCTGTTACTGGT	mCCL26-R176	TCACTGGTGCAGCTCTTGTC
hCCL27-F55	CCAGACCCTACAGCAGCATT		
hCCL27-R201	GAAAGCCTGGAGGTGACAGT		
hCCL28-F160	GCTGATGGGGATTGTGACTT	mCCL28-F226	CCGCACAATCGTACTTTGAA
hCCL28-R324	CCTGTGGCAAACATTTCCTT	mCCL28-R375	CCTGTGTGTTCCACGTGTTC
hCCR1-F571	AGCCTGAAGCAGTGGAAGAG	mCCR1-F521	CCCAATGGGAATTCACTCAC
hCCR1-R771	CAGATTGTAGGGGGTCCAGA	mCCR1-R720	CAAACGGACAGCTTTGGATT
hCCR2-F681	AAGGTTCAGGGAGCAGAGGT	mCCR2-F539	TGGCTGTGTTTGCCTCTCTA
hCCR2-F806	AGGAATTCTTCGGCCTGAGT	mCCR2-F680	TGAGGAGAAGGCAGAGCTTC
hCCR2-R1004	GGACCTCCACACACTGGTTT	mCCR2-R738	CCTACAGCGAAACAGGGTGT
hCCR2-R818	TTGCAGTCACTCAGGGAGAA	mCCR2-R828	CTCATCCTGCAGGGACAAGT
hCCR3-F445	TTTGGTGTCATCACCAGCAT	mCCR3-F295	CTGTGGAATGAGTGGGGTTT
hCCR3-R638	AGCAGAGGGAGAACGAGACA	mCCR3-R493	CAAGGCCCCAGGTAATGATA
hCCR4-F874	CTGGCTTTTGTTCACTGCTG	mCCR4-F778	GTGGTGCTTTTCCTGGAGAC
hCCR4-R1040	TAAGATGAGCTGGGGGTGTC	mCCR4-R949	GGGTGATGTACTTGCGGAAT
hCCR5-F409	TTAAAAGCCAGGACGGTCAC	mCCR5-F875	GCTGCCTAAACCCTGTCATC
hCCR5-R612	GACCAGCCCCAAGATGACTA	mCCR5-R1042	GTTCTCCTGTGGATCGGGTA
hCCR6-F929	GCCTGAACCCTGTGCTCTAC	mCCR6-F270	CCTACCGTTCTGGGCAGTTA
hCCR6-R1095	ATCTGCGGTCTCACTGGTCT	mCCR6-R438	GAAAGATTTGGTTGCCTGGA
hCCR7-F230	GGCTGGTCGTGTTGACCTAT	mCCR7-F226	AACGGGCTGGTGATACTGAC
hCCR7-R432	CATGCCACTGAAGAAGCTCA	mCCR7-R430	TCCCGCTGAAGAAGCTTAAC
hCCR8-F163	CTGGTCATCCTGGTCCTTGT	mCCR8-F529	GGCATGCTACAATGTTTCCA

Primer Name	Sequence (5 to 3)
Dominant Negative MEK1 mutant	
DNMEK1-Gibson-up	TCTGGCCTGGTCATGGCCAGAATGCTAATTCATCTGGAGATCAAA
DNMEK1-Gibson-down	TTTGATCTCCAGATGAATTAGCATTCTGGCCATGACCAGGCCAGA
Constitutively Active MEK1 mutant	
ActMEK1-Gibson-up	GGGCAGCTCATCGACGAGATGGCCAACGATTTCGTGGGCACAAGGTCC
ActMEK1-Gibson-down	GGACCTTGTGCCCACGAAATCGTTGGCCATCTCGTCGATGAGCTGCCC
pLJM1-RFP-MEK1	
EGFP-DsRED2_fwd2	tagcgctaccggtcgccaccATGGCCTCCTCCGAGGACGT
hMEK1-DsRED2_rev2	gtcggcttcttcttgggcatGGCGCCGGTGGAGTGGCGGC
pLJM1-hMEK1_rev	gctcgagatctgagtccggaTTAGACGCCAGCAGCATGGG
DsRED2-hMEK1_fwd2	gccgccactccaccggcgccATGCCCAAGAAGAAGCCGAC
pLJM1-DsRED2-hMEK1-1095F	cccatgctgctggcgtctaaTCCGGACTCAGATCTCGAGCTCAAG
pLJM1-EGFP-DsRED2-377R2	acgtcctcggaggaggccatGGTGGCGACCGGTAGCGCTA
